# Comprehensive analysis of the skeletal phenotype in *Chst14*^*−/−*^ mice: implications for dermatan sulfate in bone structure and strength

**DOI:** 10.1093/glycob/cwag037

**Published:** 2026-05-15

**Authors:** Yuki Takahashi, Takahiro Yoshizawa, Shuji Mizumoto, Fumiko Ono, Masashi Uehara, Takafumi Watanabe, Shin Shimada, Yuko Nitahara-Kasahara, Shuhei Yamada, Jun Takahashi, Takashi Okada, Tomoki Kosho

**Affiliations:** Department of Medical Genetics, Shinshu University School of Medicine, 3-1-1 Asahi, Matsumoto, Nagano 390-8621, Japan; Division of Clinical Sequencing, Shinshu University School of Medicine, 3-1-1 Asahi, Matsumoto, Nagano 390-8621, Japan; Division of Animal Research, Research Center for Advanced Science and Technology, Shinshu University, 3-1-1 Asahi, Matsumoto, Nagano 390-8621, Japan; Department of Pathobiochemistry, Faculty of Pharmacy, Meijo University, 150 Yagotoyama, Tempaku-ku, Nagoya, Aichi 468-8503, Japan; Department of Medical Genetics, Shinshu University School of Medicine, 3-1-1 Asahi, Matsumoto, Nagano 390-8621, Japan; Department of Orthopaedic Surgery, Shinshu University School of Medicine, 3-1-1 Asahi, Matsumoto, Nagano 390-8621, Japan; Laboratory of Veterinary Anatomy, School of Veterinary Medicine, Rakuno Gakuen University, 582, Bunkyodai-Midorimachi, Ebetsu, Hokkaido 069-8501, Japan; Division of Animal Research, Research Center for Advanced Science and Technology, Shinshu University, 3-1-1 Asahi, Matsumoto, Nagano 390-8621, Japan; Division of Molecular and Medical Genetics, Center for Gene and Cell Therapy, The Institute of Medical Science, The University of Tokyo, 4-6-1 Shirokanedai, Minato-city, Tokyo 108-8639, Japan; Department of Pathobiochemistry, Faculty of Pharmacy, Meijo University, 150 Yagotoyama, Tempaku-ku, Nagoya, Aichi 468-8503, Japan; Department of Orthopaedic Surgery, Shinshu University School of Medicine, 3-1-1 Asahi, Matsumoto, Nagano 390-8621, Japan; Division of Molecular and Medical Genetics, Center for Gene and Cell Therapy, The Institute of Medical Science, The University of Tokyo, 4-6-1 Shirokanedai, Minato-city, Tokyo 108-8639, Japan; Department of Medical Genetics, Shinshu University School of Medicine, 3-1-1 Asahi, Matsumoto, Nagano 390-8621, Japan; Division of Clinical Sequencing, Shinshu University School of Medicine, 3-1-1 Asahi, Matsumoto, Nagano 390-8621, Japan; Center for Medical Genetics, Shinshu University Hospital, 3-1-1 Asahi, Matsumoto, Nagano 390-8621, Japan; Research Center for Advanced Science and Technology, 3-1-1 Asahi, Shinshu University, Matsumoto, Nagano 390-8621, Japan; BioBank Shinshu, Shinshu University Hospital, 3-1-1 Asahi, Matsumoto, Nagano 390-8621, Japan

**Keywords:** bone metabolism, carbohydrate sulfotransferase 14 (CHST14), dermatan sulfate, musculocontractural Ehlers–Danlos syndrome (mcEDS)

## Abstract

Dermatan sulfate (DS) is a glycosaminoglycan known to contribute to tissue strength through collagen fibril assembly. Musculocontractural Ehlers–Danlos syndrome (mcEDS) caused by pathogenic variants in the gene that encodes carbohydrate sulfotransferase 14 (*CHST14*) (mcEDS-*CHST14*) is a representative disorder of DS deficiency. Skeletal lesions, such as progressive spinal deformity and osteoporosis/osteopenia, are significant manifestations of mcEDS-*CHST14*, which can lead to deterioration in quality of life. To characterize skeletal alterations associated with DS deficiency, we performed comprehensive analyses of the skeletal phenotypes in *Chst14* gene-deleted (*Chst14^−/−^*) mice. *Chst14^−/−^* mice exhibited progressive kyphosis, proximal femoral deformities, and decreased bone strength, as well as reduced trabecular bone mass and structural parameters, from a young age. Qualitative ultrastructural alterations of collagen fibrils in cortical bone were observed in middle-aged *Chst14^−/−^* mice by transmission electron microscopy. In addition, cortical bone showed increased expression of receptor activator of NF-κB (*Rank*), a gene involved in osteoclast differentiation, in middle age. Histomorphometric analyses of cancellous bone demonstrated reduced trabecular structural parameters in *Chst14^−/−^* mice, including decreases in bone volume and trabecular number. These findings indicate age-dependent alterations in both cortical and cancellous bone compartments that may contribute to progressive skeletal deformation. The present study provides insight into skeletal alterations associated with mcEDS-*CHST14* and highlights a broader role of DS in maintaining bone structure and strength.

## Introduction

Musculocontractural Ehlers–Danlos syndrome (mcEDS) is a subtype of EDS caused by generalized depletion of dermatan sulfate (DS). This syndrome is associated with biallelic loss-of-function variants in *CHST14*, which encodes dermatan 4-*O*-sulfotransferase 1 (D4ST1) (mcEDS-*CHST14*), or in *DSE*, which encodes DS epimerase 1 (mcEDS-*DSE*) (Dündar et al. 2009; Miyake et al. 2010; Müller et al. 2013; Malfait et al. 2017). Typical features of patients with mcEDS include various congenital abnormalities (e.g. multiple congenital joint contractures, including adducted thumbs and clubfoot, malformations involving internal organs or eyes) and progressive connective tissue fragility-related complications (e.g. hypermobility of small joints, recurrent dislocations, progressive deformities, skin hyperextensibility, fragility and bruising, large subcutaneous hematomas, and diverticular perforation) (Dündar et al. 2009; Miyake et al. 2010; Müller et al. 2013; Malfait et al. 2017; Uehara et al. 2018; Isobe et al. 2022; Minatogawa et al. 2022a; Minatogawa et al. 2022b).

DS is a glycosaminoglycan (GAG), a linear polysaccharide chain composed of repeating disaccharide units covalently attached to a core protein. Its biosynthesis begins with formation of a common tetrasaccharide linker region, GlcAβ1–3Galβ1–3Galβ1–4Xylβ1-*O*-Ser, on specific serine residues of core proteins (Kitagawa et al. 1998; Okajima et al. 1999; Gotting et al. 2000; Bai et al. 2001) (GlcA, glucuronic acid; Gal, galactose; Xyl, xylose), followed by the addition of GlcA and *N*-acetylgalactosamine (GalNAc) units to form chondroitin. Whereas chondroitin sulfate (CS) is synthesized through 4-*O*-sulfation of GalNAc catalyzed by chondroitin 4-*O*-sulfotransferase 1 (C4ST1), DS is synthesized through epimerization of chondroitin catalyzed mainly by DSE to form dermatan consisting of iduronic acid and GalNAc, followed by mostly 4-*O*-sulfation of GalNAc by D4ST1 (Evers et al. 2001; Mikami et al. 2003; Maccarana et al. 2006; Pacheco et al. 2009). D4ST1 also prevents back-epimerization of the iduronic acid–GalNAc sequence (Mizumoto and Yamada 2021). DS is either absent or markedly reduced in skin fibroblasts or urine samples of patients with mcEDS-*CHST14* or mcEDS-*DSE* (Müller et al. 2013; Janecke et al. 2016; Mizumoto et al. 2017; Lautrup et al. 2020; Minatogawa et al. 2022a). In control skin, the GAG chains of decorin, a representative DS-proteoglycan, consist almost entirely of DS, whereas in affected skin they are completely replaced by CS (Miyake et al. 2010). Unlike the round GAG chains that wrap collagen fibrils in control samples, linear CS chains interfere with proper collagen fibril assembly and lead to disorganized collagen networks (Hirose et al. 2019; Takahashi et al. 2025). mcEDS was the first reported human disorder affecting DS biosynthesis (Kosho 2016).

Progressive foot and spinal deformities can significantly impair activities of daily living and quality of life in patients with mcEDS (Kosho 2016; Uehara et al. 2018; Uehara et al. 2020; Isobe et al. 2022). In the first international collaborative study on mcEDS-*CHST14*, comprehensive information of skeletal manifestations was described including characteristic finger morphologies, joint hypermobility, multiple congenital contractures, progressive talipes deformities, and recurrent joint dislocations (Minatogawa et al. 2022b). In that report, the median age at the first dislocation was 6 years, and 80% of patients experienced an initial dislocation by 10 years of age (Minatogawa et al. 2022b). A large single-institution study of spinal lesions reported scoliosis, progressive thoracolumbar kyphosis, cervical kyphosis with vertebral malformations, and decreased bone mineral density accompanied by elevated bone resorption markers (Uehara et al. 2018). A companion study of upper limb lesions documented recurrent shoulder and elbow dislocations, osteoarthritis of the elbows and distal radioulnar joints, thin phalanges and metacarpals, and a decreased medullary cavity ratio of metacarpal bones with age (Isobe et al. 2022).

*Chst14^−/−^* mice, generated by deletion of the *Chst14* gene, have been used to study the effects of DS deficiency in the skin, urine, placenta, and skeletal muscle (Yoshizawa et al. 2018; Hirose et al. 2021; Nitahara-Kasahara et al. 2021; Yoshizawa and Kosho 2023). These mice exhibit phenotypes similar to those of patients with mcEDS, including growth impairment, skin fragility, delayed motor development with hypotonia, and spinal deformities (Akyüz et al. 2013; Yoshizawa et al. 2018; Hirose et al. 2021; Nitahara-Kasahara et al. 2021; Nitahara-Kasahara et al. 2021; Yoshizawa and Kosho 2023). Consistent with findings in patient skin samples, decorin GAG chains in *Chst14^−/−^* mouse skin consist of CS and are linear, disrupting collagen fibril assembly; by contrast, decorin in wild type (WT) skin carries DS chains that are round and wrap collagen fibrils, supporting tissue integrity (Miyake et al. 2010; Hirose et al. 2019; Hirose et al. 2021; Takahashi et al. 2025). Although bone is also a collagen-rich tissue (Lin et al. 2020), thoracolumbar kyphosis in 1-year-old mice generated using CRISPR/Cas9-mediated editing is the only bone manifestation reported to date in a mouse model of mcEDS (Nitahara-Kasahara et al. 2021), and it remains to be clarified how DS deficiency influences the progression of bone deformities from youth to middle age and how it affects osteogenesis and/or bone resorption in humans and mice. Therefore, in the present study, we investigated the role of DS in bone through a comprehensive longitudinal analysis using *Chst14^−/−^* mice. Female mice were used because the study was designed to reveal skeletal consequences of DS deficiency, including age-related effects recognized in patients with mcEDS-*CHST14* (Minatogawa et al. 2022b), while excluding sex-related effects that have not been described in patients to date. Proximal femoral deformation, progressive kyphosis, and decreased bone strength and density from a young age were observed in these mice. Structural analyses further revealed early alterations in trabecular bone architecture and age-associated changes in cortical bone structure. These findings indicate age- and compartment-specific skeletal alterations associated with DS deficiency that may contribute to progressive skeletal fragility.

## Results

### Reduced body weight gain

**Figure 1:**
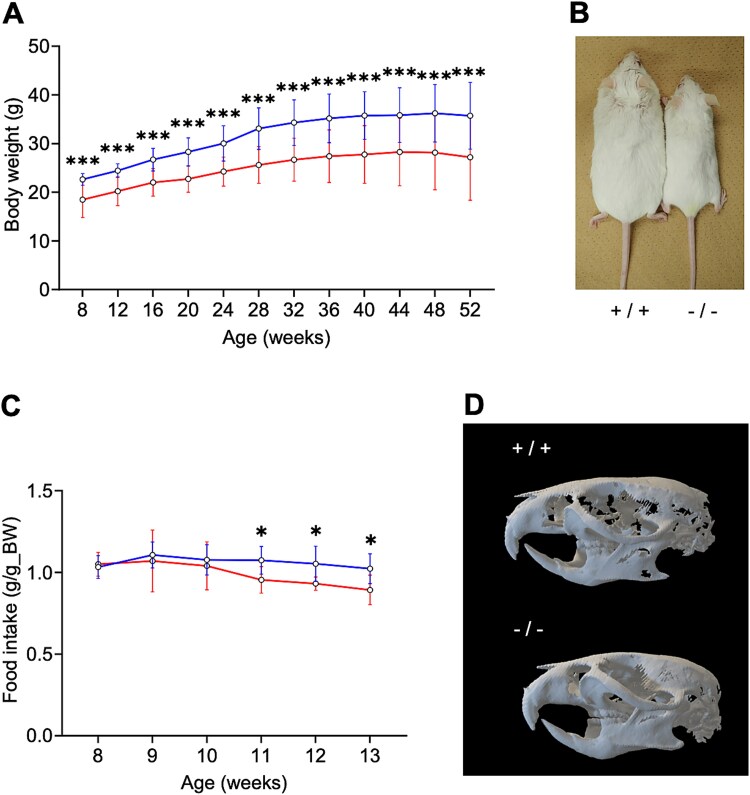
Phenotypic characterization of *Chst14^−/−^* mice. *Chst14^−/−^* mice exhibited reduced body weight from a young age, prior to decreased food intake, compared with WT mice. A) Body weight (g) of WT and *Chst14^−/−^* mice between 8 and 52 weeks of age (mean ± SD, WT: *n* = 15; *Chst14^−/−^*: *n* = 12). Blue and red lines represent WT and *Chst14^−/−^* mice, respectively. B) Appearance of 52-week-old WT (+/+) and *Chst14^−/−^* (−/−) mice. *Chst14^−/−^* mice were smaller than WT mice. C) Food intake (g) per body weight (g) of WT and *Chst14^−/−^* mice between 8 and 13 weeks of age (mean ± SD, WT: *n* = 7; *Chst14^−/−^*: *n* = 7). Blue and red lines represent WT and *Chst14^−/−^* mice, respectively. D) 3D CT images showing lateral views of the skulls of WT (+/+) and *Chst14^−/−^* (−/−) mice at 52 weeks of age. No difference in the arrangement of teeth was observed between WT and *Chst14^−/−^* mice at 52 weeks. Statistical analyses for panels a and C were performed using a two-tailed Student’s *t-*test or Welch’s two-sample *t-*test. Statistical significance is indicated as follows: ^*^P < 0.05, ^***^P < 0.001; no asterisk indicates no statistically significant difference.

The body weights of *Chst14^−/−^* mice were significantly and consistently lower than those of WT mice from 8 to 52 weeks of age (Fig. 1A). At 52 weeks, *Chst14^−/−^* mice appeared smaller than WT mice (Fig. 1B). The ratio of food intake to body weight did not differ between *Chst14^−/−^* and WT mice from 8 to 10 weeks of age; however, it was significantly reduced in *Chst14^−/−^* mice from 11 to 13 weeks (Fig. 1C). Three-dimensional (3D) computed tomography (CT) images revealed no difference in the arrangement of teeth between *Chst14^−/−^* and WT mice at 52 weeks (Fig. 1D).

### Loss of *Chst14* gene expression and absence of DS in bone tissue

**Figure 2:**
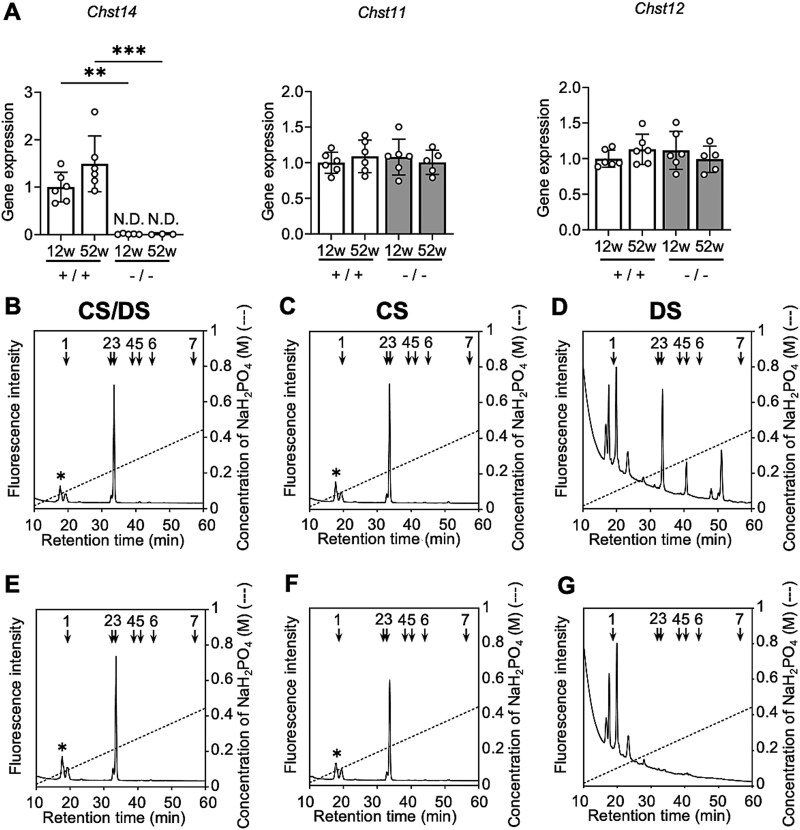
Molecular and biochemical characterization of *Chst14^−/−^* mice. *Chst14^−/−^* mice showed absent *Chst14* gene expression and DS deficiency in bone tissue. A) Relative mRNA levels of *Chst14*, *Chst11*, and *Chst12* in the femurs of WT (+/+) and *Chst14^−/−^* (−/−) mice at 12 and 52 weeks of age (mean ± SD; *n* = 6 for 12-week-old WT and *Chst14^−/−^* mice and for 52-week-old WT mice; *n* = 5 for 52-week-old *Chst14^−/−^* mice). N.D., not detected. B–G) representative anion-exchange HPLC chromatograms of tibias at 12 weeks of age are shown. These illustrate the disaccharide profiles of WT and *Chst14^−/−^* mice, revealing clear genotype-dependent differences. Corresponding quantitative data are provided in Table 1: CS/DS (B, WT; E, *Chst14^−/−^*), CS (C, WT; F, *Chst14^−/−^*), and DS (D, WT; G, *Chst14^−/−^*), obtained from digestion with chondroitinase ABC, AC, and B, respectively. Arrows indicate elution positions: 1, ΔHexA-GalNAc; 2, ΔHexA-GalNAc(6S); 3, ΔHexA-GalNAc(4S); 4, ΔHexA(2S)-GalNAc(6S); 5, ΔHexA(2S)-GalNAc(4S); 6, ΔHexA-GalNAc(4S,6S); and 7, ΔHexA(2S)-GalNAc(4S,6S). Asterisks indicate the elution position of 2AB-labeled ΔHexA- *N*-acetylglucosamine (GlcNAc) derived from hyaluronan, which is also cleaved by chondroitinases. Statistical analysis for panel a was performed using two-way ANOVA followed by Tukey’s post hoc test. Statistical significance is shown only for biologically relevant comparisons (+/+ vs −/− at the same age and 12 vs 52 weeks within genotype). Complete results of all pairwise comparisons are provided in Supplementary Table S2. Statistical significance is indicated as follows: ^**^P < 0.01, ^***^P < 0.001, ^****^P < 0.0001; no asterisk indicates no statistically significant difference.

Gene expression of *Chst14* was not detected in the femurs of *Chst14^−/−^* mice, and no significant differences were observed in the expression of *Chst11* or *Chst12* (Fig. 2A). GAG chain extracts from tibias were digested with chondroitinase ABC, AB, or B, and these digests were analyzed using HPLC to assess the amounts of CS/DS disaccharides (Fig. 2B and E), CS disaccharides (Fig. 2C and F), and DS disaccharides (Fig. 2D and G), respectively. At 12 weeks of age, the amount of CS disaccharides was not significantly different between *Chst14^−/−^* and WT mice, and DS disaccharides were not detected in the tibias of *Chst14^−/−^* mice (Table 1).

**Table 1:** Total amounts of CS and DS disaccharides (pmol/mg protein).

	CS/DS disaccharides	CS disaccharides	DS disaccharides
+/+	6518 ± 597	6159 ± 1147	132 ± 38
−/−	7903 ± 951	7273 ± 295	N.D.

Data were derived from tibias of 12-week-old WT (+/+) and *Chst14^−/−^* (−/−) mice (mean ± SD, WT: *n* = 3 [1 male and 2 female]; *Chst14^−/−^*: *n* = 3 [1 male and 2 female], N.D., not detected [<1 pmol/mg protein]). CS, chondroitin sulfate; DS, dermatan sulfate.

### Progressive spinal deformity

**Figure 3:**
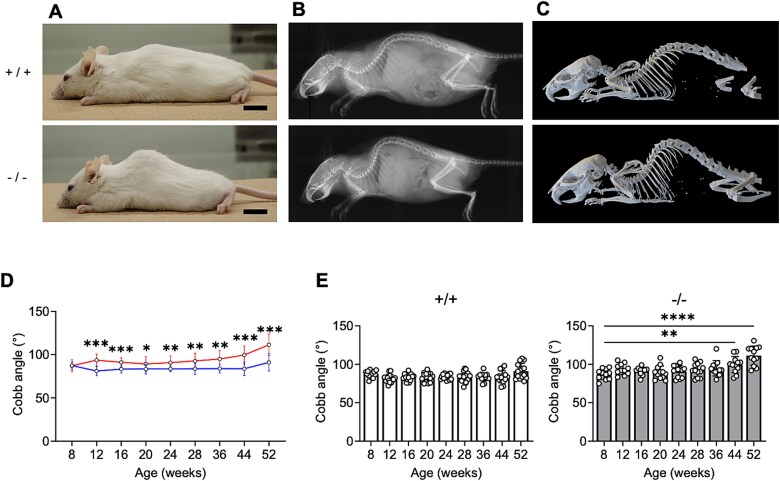
Progressive spine deformity in *Chst14^−/−^* mice. A) Lateral appearances, B) lateral radiographs, and C) 3D CT images of 52-week-old WT (+/+) and *Chst14^−/−^* (−/−) mice (scale bar: 1 cm). These panels show that *Chst14^−/−^* mice had kyphosis of the thoracolumbar junction. D) the cobb angle (indicator of the degree of kyphosis) was measured in WT (+/+) and *Chst14^−/−^* (−/−) mice from 8 to 52 weeks of age (mean ± SD, WT: *n* = 14 or 15; *Chst14^−/−^*: *n* = 11 or 12). Blue and red lines represent WT and *Chst14^−/−^* mice, respectively. E) Comparison of the cobb angle from 12 to 52 weeks of age with 8-week-old mice: WT (+/+) and *Chst14^−/−^* (−/−).*Chst14^−/−^* mice showed progressive kyphosis. Statistical analyses were performed as follows: For panel D, two-tailed Student’s *t-*test or Welch’s two-sample *t-*test; for panel E, one-way ANOVA followed by Dunnett’s post hoc test (comparison with 8-week-old mice within each genotype). Statistical significance in the graphs (D and E) is indicated as follows: ^*^P < 0.05, ^**^P < 0.01, ^***^P < 0.001, ^****^P < 0.0001; no asterisk indicates no statistically significant difference.

At 52 weeks of age, *Chst14^−/−^* mice exhibited more pronounced spinal kyphosis than WT mice, as seen in their appearance, radiographs, and 3D CT scans (Fig. 3A–C). The kyphotic Cobb angles in *Chst14^−/−^* mice were consistently and significantly greater than those in WT mice from 12 to 52 weeks of age (Fig. 3D, Fig. S1A). Comparing 8-week-old mice with older groups, *Chst14^−/−^* mice showed significantly increased kyphotic Cobb angles at 44 and 52 weeks, whereas WT mice displayed no progressive spinal deformity up to middle age (Fig. 3E).

### Deformed femoral bone from a younger age

**Figure 4:**
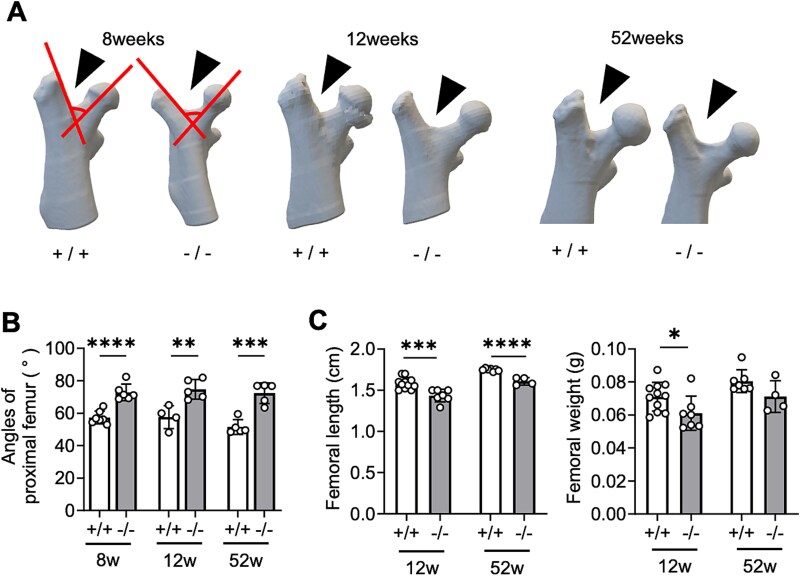
Bone deformity at proximal femurs in *Chst14^−/−^* mice. A) 3D CT images of proximal femurs from 8-, 12-, and 52-week-old WT (+/+) and *Chst14^−/−^* (−/−) mice. Arrowheads and lines indicate spaces and angles between femoral heads and greater trochanters, respectively. B) Angles between femoral heads and greater trochanters (mean ± SD, 8-week-old WT: *n* = 7; *Chst14^−/−^*: *n* = 6, 12-week-old WT: *n* = 4; *Chst14^−/−^*: *n* = 5, 52-week-old WT: *n* = 5; *Chst14^−/−^*: *n* = 5). Bone deformity appeared early in the proximal femurs of *Chst14^−/−^* mice. C) Femoral length and weight of 12-week-old WT (+/+) and *Chst14^−/−^* (−/−) mice (mean ± SD, 12-week-old WT: *n* = 11, *Chst14^−/−^*: *n* = 7; 52-week-old WT: *n* = 7, *Chst14^−/−^*: *n* = 4). Femoral length was significantly decreased in *Chst14^−/−^* mice at 12 and 52 weeks. Femoral weight was decreased at 12 weeks but not at 52 weeks. Statistical analyses for panels B and C were performed using a two-tailed Student’s *t-*test. Statistical significance in the graphs (B and C) is indicated as follows: ^*^P < 0.05, ^**^P < 0.01, ^***^P < 0.001, ^****^P < 0.0001; no asterisk indicates no statistically significant difference.

A wider space between the femoral head and the greater trochanter was observed in 3D CT images of *Chst14^−/−^* mice at 8, 12, and 52 weeks of age than in WT mice (Fig. 4A). The angle between the femoral head line and the greater trochanter line was significantly increased in *Chst14^−/−^* mice at 8, 12, and 52 weeks (Fig. 4B). Femoral length was significantly lower in *Chst14^−/−^* mice than in WT mice at 12 and 52 weeks, whereas femoral weight was significantly lower only at 12 weeks (Fig. 4C).

### Decreased strength and density of femoral bone

**Figure 5:**
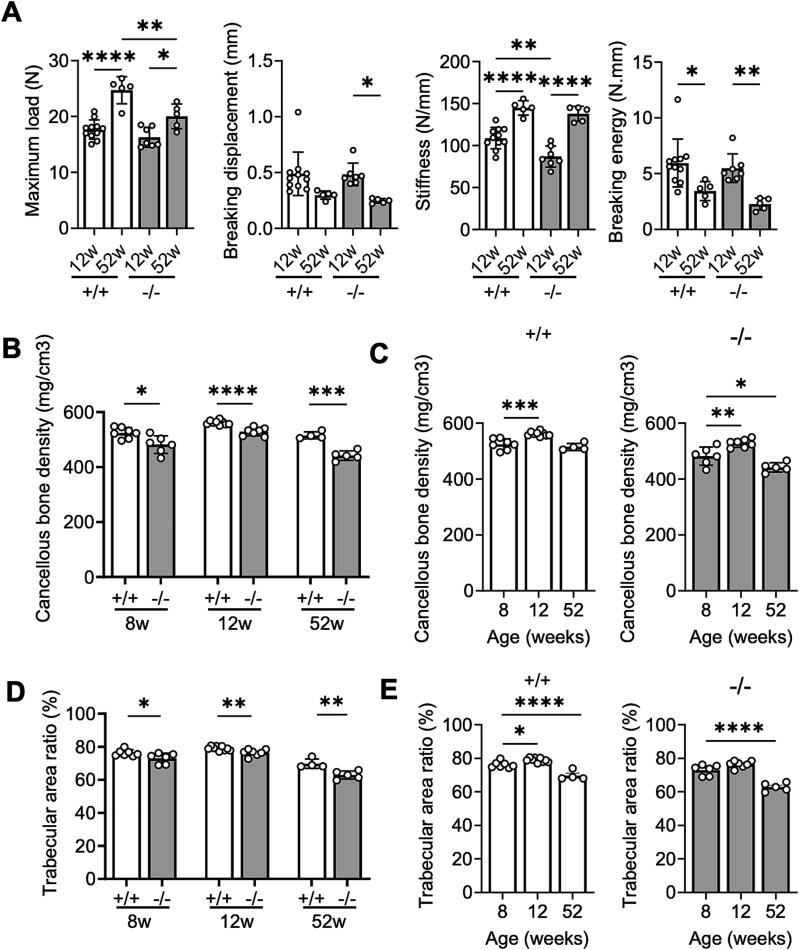
Strength and density of femurs in *Chst14^−/−^* mice. A) Femoral mid-diaphysis strength of WT (+/+) and *Chst14^−/−^* (−/−) mice evaluated through a three-point bending method (mean ± SD, 12-week-old WT: *n* = 11, *Chst14^−/−^*: *n* = 7, 52-week-old WT: *n* = 5, *Chst14^−/−^*: *n* = 5) shown with four parameters: Maximum load, breaking displacement, stiffness, and breaking energy. Femurs in *Chst14^−/−^* mice were more flexible at a young age than femurs in WT mice, but became more fragile with age. B–E) cancellous bone density and trabecular area ratio of femurs in 8-, 12-, and 52-week-old WT (+/+) and *Chst14^−/−^* (−/−) mice (mean ± SD, 8-week-old WT: *n* = 7; *Chst14^−/−^*: *n* = 6, 12-week-old WT: *n* = 9; *Chst14^−/−^*: *n* = 7, 52-week-old WT: *n* = 4; *Chst14^−/−^*: *n* = 5). Cancellous bone density and trabecular area ratio were significantly decreased in *Chst14^−/−^* mice from an early age. Statistical analyses were performed as follows: For panel a, two-way ANOVA followed by Tukey’s post hoc test; for panels B and D, two-tailed Student’s *t-*test; and for panels C and E, one-way ANOVA followed by Dunnett’s post hoc test (comparison with 8-week-old mice within each genotype). In panel a, statistical significance is shown only for biologically relevant comparisons (+/+ vs −/− at the same age and 12 vs 52 weeks within genotype). Complete results are provided in Supplementary Table S3. Statistical significance in the graphs (A–E) is indicated as follows: ^*^P < 0.05, ^**^P < 0.01, ^***^P < 0.001, ^****^P < 0.0001; no asterisk indicates no statistically significant difference.

A three-point bending test was performed on the extracted femurs to assess bone strength (Fig. 5A). The maximum load that samples could withstand without breaking was lower in 52-week-old *Chst14^−/−^* mice than in WT mice of the same age. An age-dependent increase in maximum load was observed in both *Chst14^−/−^* and WT mice (Fig. 5A). The breaking displacement, defined as the displacement at fracture, was significantly decreased in *Chst14^−/−^* mice and tended to be decreased in WT mice at 52 weeks compared with 12 weeks (Fig. 5A). Stiffness, calculated as the load-to-displacement ratio in the elastic region, was significantly lower in 12-week-old *Chst14^−/−^* mice than in WT mice of the same age and increased with age in both groups, although no significant difference was present at 52 weeks (Fig. 5A). The breaking energy, defined as the area under the load–displacement curve up to fracture, significantly decreased with age in both *Chst14^−/−^* and WT mice (Fig. 5A).

In *Chst14^−/−^* mice, proximal femoral cancellous bone densities and trabecular area ratios were significantly lower than those in WT mice at 8, 12, and 52 weeks (Fig. 5B and D). Age-dependent changes were evaluated by comparing other ages with 8 weeks. Cancellous bone density increased at 12 weeks in both WT and *Chst14^−/−^* mice, but only *Chst14^−/−^* mice showed a significant decrease by 52 weeks (Fig. 5C). The trabecular area ratio was significantly increased at 12 weeks in WT mice but not in *Chst14^−/−^* mice and significantly decreased at 52 weeks in both groups (Fig. 5E).

### Normal biochemical and endocrinological levels

**Figure 6:**
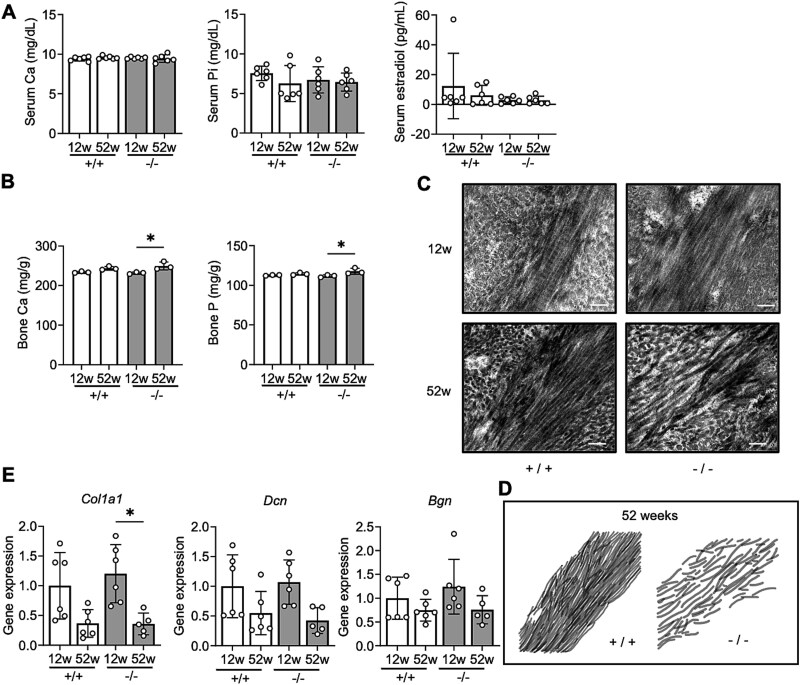
Mineral status and ultrastructural collagen features in *Chst14^−/−^* mice. A) Serum calcium (Ca), inorganic phosphate (P_i_), and estradiol concentrations (mean ± SD, each group: *n* = 6). B) Bone Ca and phosphorous (P) concentrations (mean ± SD, each group: *n* = 3). No significant differences in serum/bone mineral levels or serum estradiol levels were observed between the two genotypes. Bone mineral levels increased significantly with age only in *Chst14^−/−^* mice. C) Representative transmission electron microscopy (TEM) images showing longitudinal profiles of collagen fibrils in femoral cortical bone cross-sections from WT (+/+) and *Chst14^−/−^* (−/−) mice at 12 and 52 weeks old (scale bar: 200 nm). D) Traced images of collagen fibril networks in the femoral cortical bones of 52-week-old mice, directly derived from the TEM image shown in panel C. Age-related disorganization of collagen fibrils in the cortical bone was observed only in *Chst14^−/−^* mice. E) Relative mRNA levels of collagen type I alpha 1 (*Col1a1*), decorin (*Dcn*), and biglycan (*Bgn*) in femurs of WT (+/+) and *Chst14^−/−^* (−/−) mice at 12 and 52 weeks old (mean ± SD; *n* = 6 for 12-week-old WT and *Chst14^−/−^* mice and for 52-week-old WT mice; *n* = 5 for 52-week-old *Chst14^−/−^* mice). *Col1a1* expression decreased with age both in WT mice and *Chst14^−/−^* mice. This reduction reached statistical significance in *Chst14^−/−^* mice, whereas a non-significant trend was observed in WT mice. No significant changes in the expression of *Dcn* and *Bgn*, which encode representative DS-proteoglycan core proteins, were observed with age or between genotypes. Statistical analyses for panels A, B, and E were performed using two-way ANOVA followed by Tukey’s post hoc test. Statistical significance is shown only for biologically relevant comparisons (+/+ vs −/− at the same age and 12 vs 52 weeks within genotype). Complete results are provided in Supplementary Tables S4–S6. Statistical significance in the graphs (A, B, and E) is indicated as follows: ^*^P < 0.05; no asterisk indicates no statistically significant difference.

Serum levels of calcium (Ca), inorganic phosphate (P_i_), and estradiol did not show significant differences regardless of genotype or age (Fig. 6A). The Ca and phosphorous (P) content of femurs increased significantly with age only in *Chst14^−/−^* mice, although no significant differences were observed between WT and *Chst14^−/−^* mice of similar age (Fig. 6B).

### Age-related changes in collagen fibril ultrastructure and in expression of collagen-related genes

Transmission electron microscopy (TEM) revealed that in WT mice and in 12-week-od *Chst14^−/−^* mice, collagen fibrils in cortical bone were predominantly organized into compact and parallel bundles (Fig. 6C). By contrast, cortical bone from 52-week-old *Chst14^−/−^* mice showed areas in which the typical bundle architecture appeared partially disrupted (Fig. 6C). In these regions, fine fibrils were observed protruding from bundles, resulting in a less compact and less uniformly aligned appearance (Fig. 6C and D). Such morphological features, including fibrils protruding from bundles and irregular bundle morphology, were consistently observed across multiple fields and in the majority of 52-week-old *Chst14^−/−^* samples examined. Although mild irregularities, such as occasional waviness or crossing of fibrils, were also noted in WT mice and in 12-week-old *Chst14^−/−^* mice, these features appeared more evident in 52-week-old *Chst14^−/−^* mice. The gene expression of collagen type I alpha 1 (*Col1a1*) in the femur was significantly but modestly lower at 52 weeks than at 12 weeks in *Chst14^−/−^* mice (*P* = 0.0141) (Fig. 6E). In WT mice, *Col1a1* expression also tended to be lower at 52 weeks than at 12 weeks (*P* = 0.0639) (Fig. 6E). The gene expression of *Dcn* and *Bgn* did not show significant differences regardless of genotype or age (Fig. 6E).

### Age-related changes in bone remodeling markers

**Figure 7:**
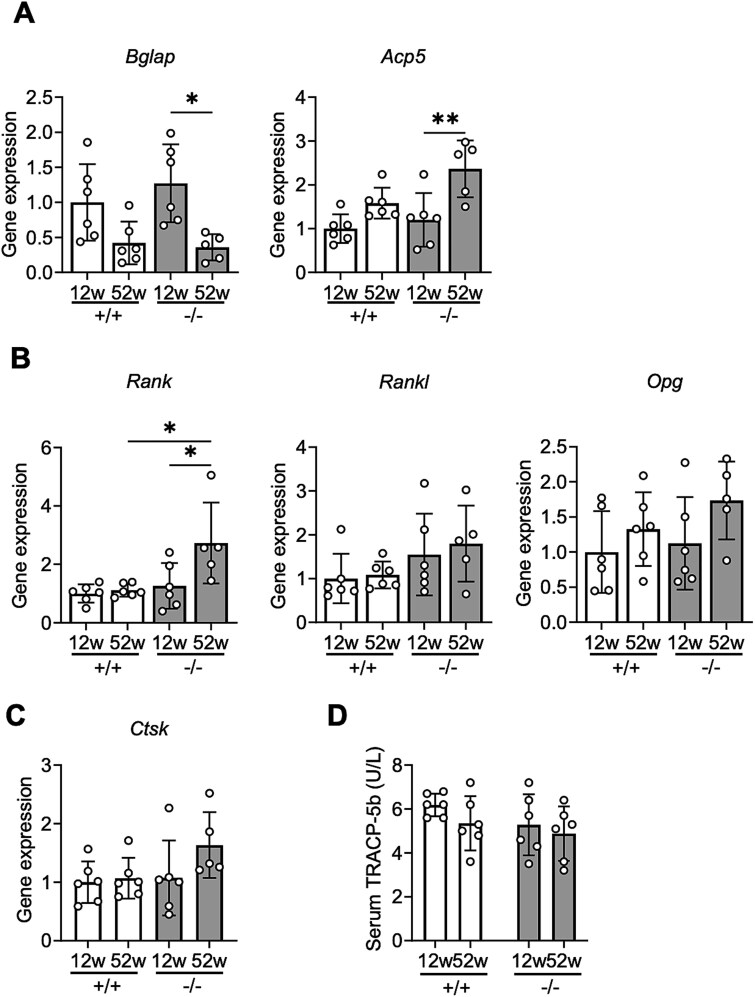
Alterations in bone metabolic factors in *Chst14^−/−^* mice. A) Relative mRNA levels of bone gamma-carboxyglutamate protein (*Bglap*) and acid phosphatase 5 (*Acp5*) in femurs of WT (+/+) and *Chst14^−/−^* (−/−) mice at 12 and 52 weeks old (mean ± SD; *n* = 6 for 12-week-old WT and *Chst14^−/−^* mice and for 52-week-old WT mice; *n* = 5 for 52-week-old *Chst14^−/−^* mice). B) Relative mRNA levels of receptor activator of NF-κB (*Rank*), receptor activator of NF-κB ligand (*Rankl*), and osteoprotegerin (*Opg*) in femurs of WT (+/+) and *Chst14^−/−^* (−/−) mice at 12 and 52 weeks old (mean ± SD; *n* = 6 for 12-week-old WT and *Chst14^−/−^* mice and for 52-week-old WT mice; *n* = 5 for 52-week-old *Chst14^−/−^* mice). Two-way ANOVA followed by Tukey’s post hoc test. C) Relative mRNA levels of cathepsin K (*Ctsk*) in femurs of WT (+/+) and *Chst14^−/−^* (−/−) mice at 12 and 52 weeks old (mean ± SD; *n* = 6 for 12-week-old WT and *Chst14^−/−^* mice and for 52-week-old WT mice; *n* = 5 for 52-week-old *Chst14^−/−^* mice). D) Serum concentrations of tartrate-resistant acid phosphatase 5b (TRACP-5b), a marker of bone resorption (mean ± SD, each group: *n* = 6). In *Chst14^−/−^* mice, gene expression analysis of cortical bone showed age-dependent changes in bone formation and resorption markers, as well as increased expression of an osteoclast differentiation factor. By contrast, the serum osteoclast marker did not change. Statistical analyses for panels A–D were performed using two-way ANOVA followed by Tukey’s post hoc test. Statistical significance is shown only for biologically relevant comparisons (+/+ vs −/− at the same age and 12 vs 52 weeks within genotype). Complete results are provided in Supplementary Tables S7–S9. Statistical significance in the graphs (A–D) is indicated as follows: ^*^P < 0.05, ^**^P < 0.01; no asterisk indicates no statistically significant difference.

The gene expression of bone gamma-carboxyglutamate protein (*Bglap*; osteoblast marker) in the femur was significantly lower and that of acid phosphatase 5 (*Acp5*; osteoclast marker) was significantly higher in 52- than in 12-week-old *Chst14^−/−^* mice (Fig. 7A). No significant age-dependent increase in *Acp5* or decrease in *Bglap* was detected in WT mice. The gene expression of *Bglap* and *Acp5* was not significantly different between WT and *Chst14^−/−^* mice of the same age (Fig. 7A). The gene expression of *Rank*, a regulatory factor for osteoclast differentiation and activation, significantly increased with age in *Chst14^−/−^* mice but not in WT mice, and it was also significantly higher in *Chst14^−/−^* mice than in WT mice at 52 weeks (Fig. 7B). The gene expression of *Rankl* and *Opg* did not show significant changes regardless of genotype or age (Fig. 7B). The gene expression of cathepsin K (*Ctsk*; an enzyme involved in osteoclastic bone resorption) did not show significant changes regardless of genotype or age (Fig. 7C). Serum levels of tartrate-resistant acid phosphatase 5b (TRACP-5b) also did not show significant differences regardless of genotype or age (Fig. 7D).

### Decreased trabecular volume and number in young *Chst14*^*−/−*^ mice

**Figure 8:**
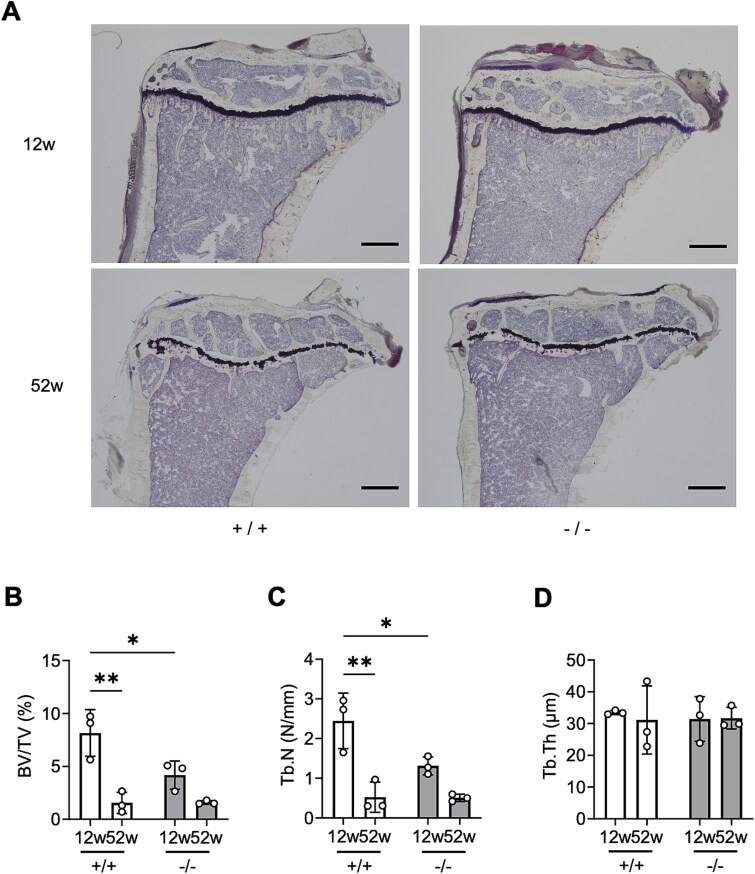
Trabecular bone structural parameters in the proximal tibia of *Chst14^−/−^* and WT mice at 12 and 52 weeks of age. A) Representative Villanueva bone–stained section of the proximal tibia. Histomorphometric parameters were quantified using Histometry RT CAMERA. Scale bar = 500 μm. B) Trabecular bone volume fraction (BV/TV, bone volume/tissue volume) in tibiae of 12- and 52-week-old WT (+/+) and *Chst14^−/−^* (−/−) mice assessed by bone histomorphometry (mean ± SD, each group: *n* = 3). C) Trabecular number (Tb.N) in tibiae of 12- and 52-week-old WT (+/+) and *Chst14^−/−^* (−/−) mice assessed by bone histomorphometry (mean ± SD, each group: *n* = 3). D) Trabecular thickness (Tb.Th) in tibiae of 12- and 52-week-old WT (+/+) and *Chst14^−/−^* (−/−) mice assessed by bone histomorphometry (mean ± SD; each group: *n* = 3). BV/TV and Tb.N were significantly decreased in 12-week-old *Chst14^−/−^* mice compared with WT mice of the same age. Statistical analyses for panels B, C, and D were performed using two-way ANOVA followed by Tukey’s post hoc test. Statistical significance is shown only for biologically relevant comparisons (+/+ vs −/− at the same age and 12 vs 52 weeks within genotype). Complete results of all pairwise comparisons are provided in Supplementary Table S10. Statistical significance in the graphs (B–D) is indicated as follows: ^*^P < 0.05, ^**^P < 0.01; no asterisk indicates no statistically significant difference.

Representative Villanueva-stained tibial sections from 12- and 52-week-old WT and *Chst14^−/−^* mice were subjected to histomorphometric analysis of trabecular bone parameters (Fig. 8A). At 12 weeks of age, bone volume (BV/TV, %) and trabecular number (Tb.N, N/mm) were significantly decreased in *Chst14^−/−^* mice compared with WT mice (Fig. 8B and C). At 52 weeks of age, BV/TV and Tb.N did not differ between genotypes (Fig. 8B and C). No differences in trabecular thickness (Tb.Th, μm) were observed across ages or between genotypes (Fig. 8D). Additional static histomorphometric parameters are presented in Supplementary Fig. S2 and Supplementary Tables S11 and S12.

## Discussion

The present study provides a comprehensive characterization of the skeletal phenotype in *Chst14^−/−^* mice, a model for mcEDS-*CHST14*, the representative human disorder caused by systemic DS deficiency. The skeletal phenotype in this mouse model included proximal femoral deformity and progressive kyphosis. Structural analyses also identified alterations in trabecular bone architecture during the early growth phase together with age-associated changes in cortical bone. These structural alterations were accompanied by reductions in bone strength, suggesting compartment-specific skeletal changes in DS-deficient mice.

Growth impairment, including significantly lower body weight and an apparently shorter body length, was observed in *Chst14^−/−^* mice compared with WT mice, consistent with previous reports (Akyüz et al. 2013; Nitahara-Kasahara et al. 2021). Because food intake did not differ between genotypes from 8 to 10 weeks of age, depletion of DS may affect prenatal skeletal development or growth in *Chst14^−/−^* mice. A previous study demonstrated significantly shorter crown–rump lengths in fetuses of *Chst14^−/−^* than of WT, consistent with our current results (Yoshizawa et al. 2018). Although food intake was reduced in *Chst14^−/−^* mice after 11 weeks of age, this decrease may have been secondary to the reduced body weight because the difference in body weight was already evident before the onset of decreased food intake. Significant growth impairment is not considered a consistent feature of mcEDS-C*HST14* (Minatogawa et al. 2022b; Kosho et al. 2025), although mild impairment was suggested in an early review (Shimizu et al. 2011).

Representative skeletal findings in *Chst14^−/−^* mice in the present study included a characteristic femoral deformity and progressive kyphosis. The femoral deformity was observed in both young (8 and 12 weeks) and older (52 weeks) mice, suggesting that it may be a congenital feature. Almost all patients (98%) with mcEDS-*CHST14* exhibit multiple congenital contractures, typically including adducted thumbs and clubfeet (Dündar et al. 2009; Miyake et al. 2010; Malfait et al. 2017; Minatogawa et al. 2022b; Uehara et al. 2023), although femoral morphology at birth or in early childhood has not yet been evaluated. DS may therefore play an important role in fetal skeletal development. Kyphosis emerged by 12 weeks and worsened with age in *Chst14^−/−^* mice in the present study. A previous study reported kyphosis only in 1-year-old mice generated using CRISPR/Cas9-mediated editing (Nitahara-Kasahara et al. 2021). Most patients (87%) with mcEDS-*CHST14* exhibit spinal deformities, including progressive thoracolumbar kyphosis with or without mild scoliosis (Uehara et al. 2018; Minatogawa et al. 2022b; Uehara et al. 2023; Kosho et al. 2025). Loose ligaments and muscle hypotonia may contribute to these progressive deformities in patients with mcEDS-*CHST14*, as well as in *Chst14^−/−^* mice, as has been proposed for patients with kyphoscoliosis EDS (Uehara et al. 2023). Most patients with mcEDS-*CHST14* show hypotonia (86%) and gross motor delay (87%) (Minatogawa et al. 2022b; Kosho et al. 2025), and *Chst14^−/−^* mice exhibit decreased grip strength and mild myopathy on pathological examination (Nitahara-Kasahara et al. 2021; Nitahara-Kasahara et al. 2021).

In the present study, *Chst14^−/−^* mice exhibited significantly reduced bone stiffness at a young age and reduced maximum load at middle age compared with WT mice. Maximum load was also significantly decreased at an older age compared with WT mice, whereas breaking energy decreased with age in both genotypes. These findings suggest that bones of *Chst14^−/−^* mice may be more prone to deformation at younger ages than those of WT mice. Furthermore, age-related increases in fracture susceptibility appear to occur in both genotypes, although bones of *Chst14^−/−^* mice may become more fragile with age. In addition to spinal deformities, most patients with mcEDS-*CHST14* exhibit foot and ankle deformities (98%) and pectus deformities (84%) from early ages (Uehara et al. 2023), although these features could also be influenced by fragility of surrounding tissues, including ligaments.

Cancellous bone density was also evaluated in the present study. It showed a significant decrease in young to middle-aged *Chst14^−/−^* mice compared with WT mice of the same age and a significant age-related decrease only in *Chst14^−/−^* mice. Given that no significant differences were observed between *Chst14^−/−^* and WT mice in serum and bone levels of Ca and P or in serum estradiol levels, it is unlikely that these changes reflect alterations in mineral metabolism or sex hormone balance. Patients with mcEDS-*CHST14* generally exhibit osteoporosis and/or reduced bone mineral density (74%), although an increased frequency of fractures has not been reported to date (Kosho et al. 2025). In addition, the trabecular area ratio showed a significant decrease in young to middle-aged *Chst14^−/−^* mice compared with WT mice of the same age, and an initial increase was observed only in WT mice. These findings suggest that impaired trabecular formation in young *Chst14^−/−^* mice may contribute to alterations in bone microarchitecture. After micro–computed tomography (μCT) analysis revealed alterations in trabecular bone architecture, we next examined the ultrastructural organization of collagen fibrils in cortical bone. Ultrastructural alterations of collagen fibrils, including less uniform alignment, were observed only in middle-aged *Chst14^−/−^* mice by TEM. Similar features were observed across multiple animals and tissue fields. Because the TEM analysis was qualitative in nature, these observations should be interpreted with caution; however, they suggest that loss of DS may influence collagen fibril bundle organization. Further studies will be required to clarify the underlying mechanisms. Skin fragility in patients with mcEDS-*CHST14* and in *Chst14^−/−^* mice has been shown, through comprehensive electron microscopic studies using cupromeronic blue staining to visualize decorin GAG chains, to result from impaired collagen fibril assembly and subsequent spatial disorganization of collagen networks (Hirose et al. 2019; Hirose et al. 2021; Takahashi et al. 2025). This impaired assembly was attributed to structural alterations in decorin GAG side chains due to complete replacement of DS with CS (Miyake et al. 2010; Hirose et al. 2019; Hirose et al. 2021; Takahashi et al. 2025). Although DS constitutes approximately 80% of total GAGs in skin (Hirose et al. 2021), it accounts for only 2% of total GAGs in bone (Table 1), where CS is the predominant GAG. Therefore, the apparent alterations in collagen fibril bundle organization observed in the bone of *Chst14^−/−^* mice in the present study may not simply reflect glycobiological replacement of decorin GAG chains but may instead be mediated by bone-specific mechanisms, potentially involving changes in bone remodeling processes, as discussed below.

In the present study, gene expression analyses were performed on cortical bone from the femoral diaphysis because its dense structure enables extraction of high-quality RNA. The results showed a significant decrease in the osteoblast marker *Bglap* and a significant increase in the osteoclast marker *Acp5* with age only in *Chst14^−/−^* mice. In addition, expression of the osteoclast differentiation marker *Rank* was significantly increased in middle-aged *Chst14^−/−^* mice compared with WT mice. A previous in vitro study demonstrated that DS inhibits the binding of RANK to RANKL in a quartz-crystal microbalance assay because of its high binding affinity to RANKL and suppresses osteoclast formation in a dose-dependent manner (Shinmyouzu et al. 2007). Because serum levels of the osteoclast marker TRACP-5b did not differ by age or genotype, these findings may reflect local alterations in osteoclast-related gene expression in bone tissue that are not captured by systemic serum markers. However, because the analyses were limited to mRNA-level gene expression and no functional assays were performed, the impact on actual bone resorption remains uncertain. Although these ultrastructural and gene expression findings provide potential insights into local matrix organization and remodeling activity, the major structural phenotype of bone was characterized by the histomorphometric analyses described below.

Histomorphometric analyses of cancellous bone demonstrated reduced trabecular structural parameters in *Chst14^−/−^* mice, including decreases in BV/TV and Tb.N at a young age. These findings suggest impaired trabecular structural development during the early growth phase, as the age-dependent increase in μCT-measured trabecular parameters observed in WT mice between 8 and 12 weeks was absent in *Chst14^−/−^* mice. Consistent with these histomorphometric findings, μCT analyses demonstrated reduced cancellous bone density and trabecular area ratio in *Chst14^−/−^* mice, both reflecting alterations in the trabecular compartment. In WT mice, an age-dependent increase in the trabecular area ratio between 8 and 12 weeks was observed, whereas this increase was not detected in *Chst14^−/−^* mice. A significant decrease in calcium deposition, an indicator of osteogenesis, was shown in a study using osteogenic lineages derived from induced pluripotent stem cells of patients with mcEDS-*CHST14,* suggesting impaired osteogenesis (Yue et al. 2023). In contrast to the cancellous compartment, age-associated alterations were observed in cortical bone. These included changes in osteoblast- and osteoclast-related gene expression, as well as qualitative differences in collagen fibril organization based on descriptive TEM observations without quantitative analysis. Although cancellous histomorphometry demonstrated alterations in trabecular bone structure, it did not indicate overt dysregulation of bone turnover. The cortical molecular and structural findings suggest compartment-specific differences in skeletal regulation with aging. Reductions in bone strength parameters occurred alongside these cortical alterations. Although causality cannot be established in the present study, these findings may be associated with progressive skeletal fragility arising from early alterations in trabecular structure and later cortical changes.

Limitations of the present study are summarized as follows. First, the analyses were predominantly performed on female mice. In our international collaborative clinical study on mcEDS-*CHST14* (Minatogawa et al. 2022b), which included equal numbers of male and female patients, sex-related phenotypic differences were not reported, whereas age-related progression of skeletal lesions was observed. Preliminary observations in male *Chst14^−/−^* mice showed trends similar to those in female mice (Fig. S3A–E). Therefore, a single sex (female) was used to investigate the skeletal consequences of DS deficiency while minimizing potential sex-related variability. Second, surrounding tissues such as muscle, tendon, and cartilage, which may influence bone morphology, were not evaluated in the present study. Because interactions between bone and surrounding musculoskeletal tissues can influence skeletal morphology and mechanical loading (Schoenau 2005), it is possible that the femoral deformity and progressive kyphosis observed in the present study may partially reflect alterations in these tissues, in addition to bone-intrinsic changes. Further studies examining interactions between bone and surrounding tissues will be required to clarify these mechanisms. Third, molecular analyses in the present study were limited to gene expression measurements, and corresponding protein levels or extracellular matrix deposition were not examined. Because obtaining sufficient numbers of aged specimens for longitudinal analysis was challenging, we focused on transcriptional changes. Future studies will be required to determine whether the observed changes in *Col1a1* and *Rank* expression at the mRNA level lead to alterations in collagen synthesis, extracellular matrix organization, or osteoclast-related signaling pathways.

In conclusion, this comprehensive longitudinal analysis of *Chst14^−/−^* mice, a model for DS deficiency, revealed previously unrecognized skeletal phenotypes, including proximal femoral deformation and progressive kyphosis. Structural analyses further identified early alterations in trabecular bone architecture and age-associated changes in cortical bone. Together with the observed reductions in bone strength, these findings suggest compartment-specific skeletal alterations that may contribute to progressive skeletal fragility. These results also highlight a broader role of DS in maintaining bone structure and strength. While these findings provide insight into age- and compartment-specific skeletal alterations associated with DS deficiency, further pathophysiological and translational studies will be required before therapeutic applications can be considered.

## Materials and methods

### Animals

*Chst14* gene-deleted mice with a B6;129 genetic background were obtained from the Mutant Mouse Regional Resource Center (https://www.mmrrc.org) (Tang et al. 2010). These mice exhibited an embryonic lethal phenotype, and only a limited number of adult mice were obtained (1.3%) (Yoshizawa et al. 2018). Thus, the mice were backcrossed to the BALB/cAJcl (BALB/cA) strain (CLEA Japan, Inc., Shizuoka, Japan) for 12 generations, which improved the birth rate of *Chst14^−/−^* mice to 6.12%–18.64% (Shimada et al. 2020). In the present study, BALB/c congenic *Chst14^−/−^* mice were used to facilitate efficient analysis in adult stages because they survive beyond birth unlike those with a B6;129 genetic background. The mice were housed in a microisolator (Shin Toyo Seisakusho, Kawaguchi, Japan) at 23 °C ± 3 °C with constant humidity and a 12-h light/dark cycle. The animals had free access to tap water and standard mouse chow (Funabashi Farm, Funabashi, Japan). Female mice were used throughout the study to focus on the direct skeletal effects of DS deficiency, including age-related effects recognized in patients (Minatogawa et al. 2022b). The exception to this was a preliminary part of the study involving quantification of DS and CS (Fig. 2B–D), which included one male and two female mice.

In each experimental procedure, each mouse was treated independently using the same protocol, representing a biological replicate and ensuring data consistency. All mice were bred in-house, and the number of age-matched mice used for each experimental procedure varied depending on their birth dates, as indicated in the corresponding figure legends. Similar tendencies in bone phenotypes were also observed in male mice in preliminary experiments (Fig. S3A–E).

### Genotyping PCR

PCR was performed as previously described (Yoshizawa et al. 2018; Shimada et al. 2020). Pups were weaned and ear-punched at 3 weeks of age. DNA was extracted from ear tissue using Mighty Prep reagent for DNA (Takara Bio Inc., Shiga, Japan). Primer sequences for PCR genotyping of WT, designed for exon 1 of the *Chst14* gene, were 5′-GGACCACCGCAGTGACTTG-3′ and 5′-ACAGGCATCCAATGCTCATTC-3′. Primer sequences for the neomycin resistance gene in *Chst14^−/−^* mice were 5′-TGGCTCTCCTCAAGCGTATT-3′ and 5′-GTTTTCCCAGTCACGACGTT-3′. The extracted DNA solutions were used for PCR with HS Perfect Mix (Takara Bio Inc.). The PCR conditions were 94 °C for 1 minute, followed by 30 cycles of 94 °C for 5 seconds and 65 °C for 15 seconds. PCR products were detected using agarose gel electrophoresis.

### Body weight and food intake

The body weight and food intake of WT and *Chst14^−/−^* mice were measured weekly from 8 to 52 weeks of age and from 8 to 13 weeks of age, respectively. Food intake analysis was performed on individually housed mice.

### Quantification of DS and CS

Twelve-week-old mice were euthanized with an overdose of pentobarbital sodium (150 mg/kg). Tibias were collected, with the surrounding soft tissues removed, and frozen in liquid nitrogen. The samples were homogenized and sonicated. Extraction and purification of GAG fractions were carried out as described previously (Mizumoto and Sugahara 2012). Briefly, the GAG fractions were digested with a mixture of chondroitinase ABC and AC-II, chondroitinase AC-I and AC-II, or chondroitinase B at 37 °C for 2 h. Each digest was labeled with the fluorophore 2-aminobenzamide, and the disaccharides were quantified by anion-exchange HPLC.

### Gene expression analysis

During dissection, the femoral diaphyses were separated from the metaphyseal regions and bone marrow and then frozen. The frozen diaphyses were subsequently crushed in liquid nitrogen and homogenized in TRI Reagent (Molecular Research Center, Cincinnati, OH, USA) by vortexing to extract total RNA. The homogenate was purified, and contaminating DNA was removed using the RNA Clean & Concentrator-5 kit (Zymo Research, Irvine, CA, USA). The RNA was reverse-transcribed using a High-Capacity cDNA Reverse Transcription Kit (Applied Biosystems, Foster City, CA, USA). Quantitative reverse-transcription PCR was performed using a QuantStudio® 3 Real-Time PCR system (Applied Biosystems) with THUNDERBIRD Next SYBR qPCR Mix (Toyobo, Osaka, Japan). Values were normalized to 18S ribosomal RNA levels. The primer sequences are listed in Supplementary Table S1. Primer sequences for *Bglap* and *Ctsk* were based on previous publications (Chan et al. 2018; Moriishi et al. 2020), while other primers were designed using Primer3Plus software (Whitehead Institute for Biomedical Research, Cambridge, MA, USA).

### Kyphosis cobb angle assessment

Plain radiographs were taken using a quantitative CT system (LaTheta LCT-200; Hitachi Healthcare BU, Tokyo, Japan) every 4 weeks from 8 to 28 weeks of age, and every 8 weeks thereafter until 52 weeks. A mixture of medetomidine hydrochloride (0.3 mg/kg body weight) (Nippon Zenyaku Kogyo Co., Ltd., Fukushima, Japan), midazolam (4.0 mg/kg body weight) (Sandoz K.K., Tokyo, Japan), and butorphanol tartrate (5.0 mg/kg body weight) (Meiji Seika Pharma Co., Ltd., Tokyo, Japan) was administered intraperitoneally (10 mL/kg body weight), and radiographs were taken in the right lateral decubitus position. After the radiographs were taken, atipamezole hydrochloride (0.3 mg/kg body weight) (Nippon Zenyaku Kogyo Co., Ltd.), an antagonist of medetomidine hydrochloride, was administered intraperitoneally (10 mL/kg body weight). The degree of kyphosis was evaluated using the Cobb method. The kyphosis Cobb angle at the thoracolumbar junction was measured using ImageJ from the obtained images (Schneider et al. 2012). Briefly, the angle at the intersection of the vertical lines near the inflection point of the spinal curve was measured on lateral recumbent radiographs.

### Angle of proximal femur assessment

The angle of the proximal femur was measured in excised femurs from 8-, 12-, and 52-week-old mice. The femurs were imaged using a quantitative CT system (LaTheta LCT-200; Hitachi Healthcare BU) with a pixel size of 24 μm and a slice thickness of 48 μm. 3D reconstruction of the images was performed using Volume Extractor ver.3.0 (i-Plants systems, Iwate Prefectural University, Japan) to examine the morphology of the proximal femur. The angle was measured using ImageJ (Schneider et al. 2012).

### Bone strength analysis

Twelve- and 52-week-old mice were euthanized with an overdose of pentobarbital sodium (150 mg/kg), and the femurs were collected with surrounding soft tissue removed. The three-point bending test was performed at a speed of 2 mm/min with a distance of 6 mm between fulcrums, conducted by Kureha Special Laboratory Co., Ltd. (Fukushima, Japan).

### Histomorphometry of tibial cancellous bone

After the mice were euthanized by cervical dislocation, tibias were collected and fixed in 70% ethanol. The samples were stained with Villanueva Bone Stain and embedded in methyl methacrylate (Wako Pure Chemical Industries, Ltd., Osaka, Japan). Histomorphometric measurements in the secondary spongiosa beneath the growth plate were performed at the Niigata Bone Science Institute (Niigata, Japan).

### Bone mineral density measurements

Bone mineral density analyses were performed using a quantitative CT system (LaTheta LCT-200; Hitachi Healthcare BU). Briefly, the area from the tip of the femoral head to immediately below the greater trochanter of the excised femur was imaged with a pixel size of 24 μm and a slice thickness of 48 μm. The values for cancellous bone density and trabecular area ratio were calculated using LaTheta software ver. 3.6 (Hitachi Healthcare BU).

### Blood measurements

Blood samples were collected from 12- and 52-week-old mice via the abdominal vena cava, and serum was separated using Bloodsepar (IBL, Gunma, Japan). Serum Ca, P_i_, estradiol, and TRACP-5b were measured by Oriental Yeast Co., Ltd. (Tokyo, Japan).

### Bone mineral content analysis

After the mice were euthanized by cervical dislocation, tibias were collected and fixed in 70% ethanol. Ca and P levels in bone were measured using inductively coupled plasma emission spectrometry, performed by Kureha Special Laboratory Co., Ltd. (Fukushima, Japan).

### TEM

Mice aged 12 and 52 weeks were anesthetized with 3% isoflurane. After perfusion fixation with 2.5% glutaraldehyde, femoral bones were collected, soft tissues were removed, and the bones were fixed in 2.5% glutaraldehyde for 1 to 2 weeks at 4 °C. The samples were defatted with ethanol and then decalcified using EDT-X (Falma, Tokyo, Japan). The decalcified samples were cut into pieces with a scalpel, refixed in 2.5% glutaraldehyde at 4 °C overnight, postfixed in 1.0% osmium tetroxide, dehydrated in graded ethanol, and transferred to propylene oxide (Nisshin EM, Tokyo, Japan). The samples were embedded in epoxy resin (Okenshoji, Tokyo, Japan), polymerized, and cut into ultrathin sections using an ULTRACUT R (Leica, Wetzlar, Germany) equipped with a diamond knife. Ultrathin sections, 60–70 nm thick, were mounted on copper grids and stained with 0.2% oolong tea extract (Nisshin EM). The sections were then further stained with uranyl acetate and lead citrate. Finally, the sections were subjected to carbon shadowing and observed by TEM (JEM1400; JEOL, Tokyo, Japan) at an accelerating voltage of 80 kV. Collagen fibril bundle morphology was qualitatively assessed in longitudinal profiles of collagen fibrils observed by TEM in femoral cortical bone cross-sections. The assessment focused on morphological features such as fibrils protruding from bundles and regions where the bundle architecture appeared less compact compared with the well-organized bundles typically observed in WT cortical bone. No quantitative measurements were performed.

For each experimental group, samples from three animals were examined, and at least five tissue fields within the cortical bone were analyzed per sample.

### Statistical analysis

Data are presented as mean ± standard deviation (SD). Statistical comparisons between two groups were performed using the unpaired two-tailed Student’s *t-*test or Welch’s *t-*test. Multiple comparisons between three or more groups were performed using one-way or two-way analysis of variance (ANOVA), followed by Tukey’s or Dunnett’s multiple comparisons test. Tukey’s multiple comparison test was performed, but only predefined contrasts (genotype differences within age groups and age differences within genotype) are shown in the main figures; complete results are reported in Supplementary Tables S2–S11. Statistical significance was defined as *P* < 0.05, and all analyses were performed using EZR (Jichi Medical University, Shimotsuke, Japan) (Kanda 2013) and Prism 9 or 10 (GraphPad, La Jolla, CA, USA).

### Study approval

All experimental procedures were reviewed and approved by the Committee for Animal Experiments, under the approval of the President of Shinshu University (Approval numbers 019048 and 021121), in accordance with national regulations and guidelines. All procedures were conducted in compliance with the Regulations for Animal Experimentation of Shinshu University.

## Supplementary Material

Supplementary_matrials_cwag037

## Data Availability

All data are provided in the figures, tables, and supplementary data in this manuscript.
