# Supplementary material for: Comprehensive analysis of the skeletal phenotype in Chst14−/− mice: implications for dermatan sulfate in bone structure and strength
Source: Glycobiology. 2026 May 15;36(7):cwag037. doi: 10.1093/glycob/cwag037 (PMC13196589; doi:10.1093/glycob/cwag037)
Supplement: Supplementary_matrials_cwag037 [file supplementary_matrials_cwag037.zip › Supplementary Table S12 (Glyco Revise).pdf]

Table S12. Tukey's multiple comparisons test (Figure S2B)

Ob.S/BS (%)

Comparison	Predicted (LS) mean diff.	95.00% CI of diff.	Adjusted P Value
12w:+/+ vs. 12w:-/-	-10.23	-23.56 to 3.102	0.1427
12w:+/+ vs. 52w:+/+	12.97	-0.3642 to 26.29	0.0566
12w:+/+ vs. 52w:-/-	7.125	-6.205 to 20.45	0.3778
12w:-/- vs. 52w:+/+	23.19	9.863 to 36.52	0.0024
12w:-/- vs. 52w:-/-	17.35	4.023 to 30.68	0.0133
52w:+/+ vs. 52w:-/-	-5.84	-19.17 to 7.489	0.5312

OS/BS (%)

Comparison	Predicted (LS) mean diff.	95.00% CI of diff.	Adjusted P Value
12w:+/+ vs. 12w:-/-	-10.56	-30.15 to 9.024	0.371
12w:+/+ vs. 52w:+/+	8.82	-10.77 to 28.41	0.5103
12w:+/+ vs. 52w:-/-	-1.847	-21.43 to 17.74	0.9897
12w:-/- vs. 52w:+/+	19.38	-0.2036 to 38.97	0.0524
12w:-/- vs. 52w:-/-	8.715	-10.87 to 28.30	0.5195
52w:+/+ vs. 52w:-/-	-10.67	-30.25 to 8.919	0.3635

OV/BV (%)

Comparison	Predicted (LS) mean diff.	95.00% CI of diff.	Adjusted P Value
12w:+/+ vs. 12w:-/-	-5.164	-9.643 to -0.6856	0.0253
12w:+/+ vs. 52w:+/+	3.45	-1.029 to 7.928	0.1408
12w:+/+ vs. 52w:-/-	1.639	-2.839 to 6.118	0.6592
12w:-/- vs. 52w:+/+	8.614	4.135 to 13.09	0.0012
12w:-/- vs. 52w:-/-	6.804	2.325 to 11.28	0.0055
52w:+/+ vs. 52w:-/-	-1.81	-6.289 to 2.668	0.5908

O.Th (μm)

Comparison	Predicted (LS) mean diff.	95.00% CI of diff.	Adjusted P Value
12w:+/+ vs. 12w:-/-	-0.958	-2.036 to 0.1200	0.0827
12w:+/+ vs. 52w:+/+	1.514	0.4364 to 2.592	0.0087
12w:+/+ vs. 52w:-/-	1.325	0.2467 to 2.403	0.0182
12w:-/- vs. 52w:+/+	2.472	1.394 to 3.550	0.0004
12w:-/- vs. 52w:-/-	2.283	1.205 to 3.361	0.0006
52w:+/+ vs. 52w:-/-	-0.1897	-1.268 to 0.8883	0.9402
