# Supplementary material for: Comprehensive analysis of the skeletal phenotype in Chst14−/− mice: implications for dermatan sulfate in bone structure and strength
Source: Glycobiology. 2026 May 15;36(7):cwag037. doi: 10.1093/glycob/cwag037 (PMC13196589; doi:10.1093/glycob/cwag037)
Supplement: Supplementary_matrials_cwag037 [file supplementary_matrials_cwag037.zip › Supplementary_Figure_S1_20260419.pdf]

A

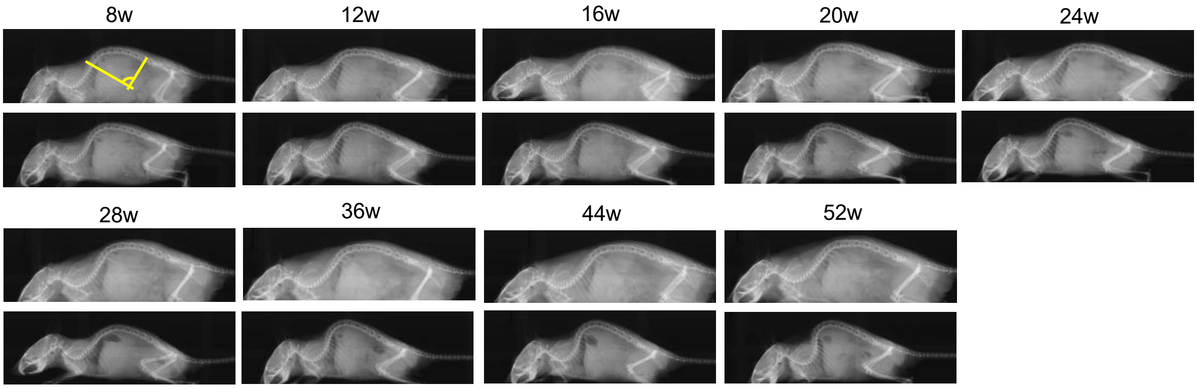

Figure S1. Serial radiographs for assessment of the degree of kyphosis. A) Lateral radiographs showing kyphotic changes from 8 to 52 weeks of age in WT (+/+) and *Chst14*$^{-/-}$ (−/−) mice. The Cobb angle was measured between the yellow lines: one drawn along the upper endplate of the uppermost tilted vertebra and the other along the lower endplate of the lowermost tilted vertebra.
