# Supplementary material for: Comprehensive analysis of the skeletal phenotype in Chst14−/− mice: implications for dermatan sulfate in bone structure and strength
Source: Glycobiology. 2026 May 15;36(7):cwag037. doi: 10.1093/glycob/cwag037 (PMC13196589; doi:10.1093/glycob/cwag037)
Supplement: Supplementary_matrials_cwag037 [file supplementary_matrials_cwag037.zip › Supplementary_Figure_S2_20260419.pdf]

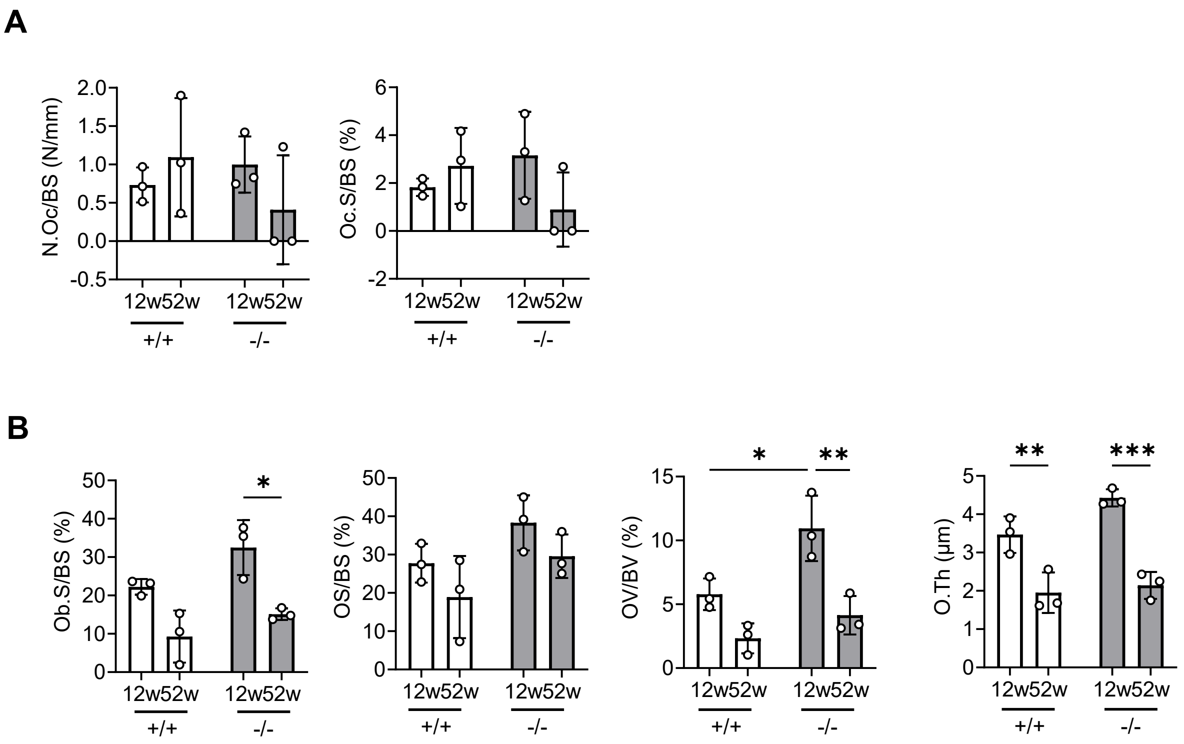

Figure S2. Osteoclast and osteoblast numbers in tibial cancellous bone of *Chst14*$^{-/-}$ and WT mice at young and middle age. A) Bone resorption markers in tibiae of 12- and 52-week-old WT (+/+) and *Chst14*$^{-/-}$ (−/−) mice assessed by bone histomorphometry (mean ± SD, each group: n = 3). N.Oc/BS, osteoclast number/bone surface; Oc.S/BS, osteoclast surface/bone surface. Two-way ANOVA followed by Tukey's post hoc test. B) Bone formation markers in tibiae of 12- and 52-week-old WT (+/+) and *Chst14*$^{-/-}$ (−/−) mice assessed by bone histomorphometry (mean ± SD, each group: n = 3). Ob.S/BS, osteoblast surface/bone surface; OS/BS, osteoid surface/bone surface; OV/BV, osteoid volume/bone volume; O.Th, osteoid thickness. Two-way ANOVA followed by Tukey's post hoc test. Statistical significance in the graphs (A and B) is indicated as follows: *$P < 0.05$, **$P < 0.01$, ***$P < 0.001$; no asterisk indicates no statistically significant difference. Statistical significance is shown only for biologically relevant comparisons (+/+ vs −/− at the same age and 12 vs 52 weeks within genotype). Complete results of all pairwise comparisons are provided in Supplementary Tables S11 and S12.
