# Supplementary material for: Comprehensive analysis of the skeletal phenotype in Chst14−/− mice: implications for dermatan sulfate in bone structure and strength
Source: Glycobiology. 2026 May 15;36(7):cwag037. doi: 10.1093/glycob/cwag037 (PMC13196589; doi:10.1093/glycob/cwag037)
Supplement: Supplementary_matrials_cwag037 [file supplementary_matrials_cwag037.zip › Supplementary_Figure_S3_20260419.pdf]

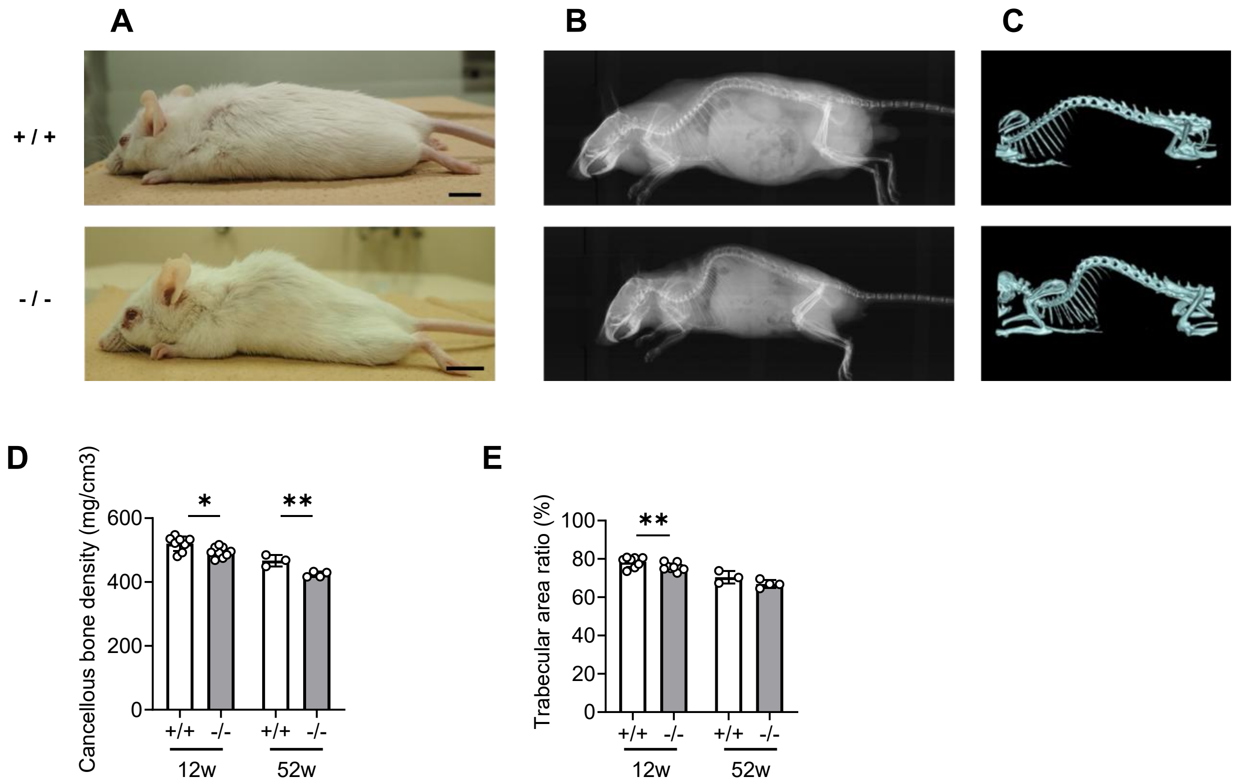

Figure S3. Bone phenotype in male *Chst14*$^{-/-}$ mice. In *Chst14*$^{-/-}$ mice, male mice exhibited a bone phenotype similar to that of female mice. A) Lateral appearances, B) lateral radiographs, and C) 3D CT images of 52-week-old WT (+/+) and *Chst14*$^{-/-}$ (−/−) male mice (scale bar: 1 cm). These panels show that *Chst14*$^{-/-}$ mice had kyphosis of the thoracolumbar junction. D and E, Cancellous bone density and trabecular area ratio of femurs in 12- and 52-week-old WT (+/+) and *Chst14*$^{-/-}$ (−/−) mice (mean ± SD, 12-week-old WT: n = 8; *Chst14*$^{-/-}$: n = 9, 52-week-old WT: n = 3; *Chst14*$^{-/-}$: n = 4). Cancellous bone density and trabecular area ratio were significantly decreased in *Chst14*$^{-/-}$ mice from an early age, except for the trabecular area ratio in 52-week-old male mice, which showed no significant difference. Two-tailed Student's t-test. Statistical significance in the graphs (D and E) is indicated as follows: $*P < 0.05$, $**P < 0.01$; no asterisk indicates no statistically significant difference.
