# Supplementary material for: Comprehensive analysis of the skeletal phenotype in Chst14−/− mice: implications for dermatan sulfate in bone structure and strength
Source: Glycobiology. 2026 May 15;36(7):cwag037. doi: 10.1093/glycob/cwag037 (PMC13196589; doi:10.1093/glycob/cwag037)
Supplement: Supplementary_matrials_cwag037 [file supplementary_matrials_cwag037.zip › Supplementary_Table_S1_(Glyco_Revise).pdf]

Table S1. Primer sequencing quantitative PCR analysis

Gene	Sequence (5'–3')
18s	ATCATGCAGAACCCACGACA
	ACACGAAGGCCCCAAAAGT
Chst14	CCAAAGTGGCCTGCTCTAACTG
	AAGTCACTGCGGTGGTCCAT
Chst11	CCCTTCGGTGTGGACATCTG
	GCAGGATGGCAGTGTTGGAT
Chst12	TTCTGACGCAAAGCACAACG
	AGGGTTCTGATGGGCATCATG
Col1a1	ATGGATTCCCGTTCGAGTACG
	TCAGCTGGATAGCGACATCG
Dcn	ATCACCAAGCTGCGGAAATC
	AAGGCTCCGTTTTCAATCCC
Bgn	GAGGGAACTTCACTTGGACAAC
	CACCTTGGTGATGTTGTTGG
Bglap	CCTGGCTGCGCTCTGTCT
	TGCTTGGACATGAAGGCTTTG
Acp5	GCTTCTCTGCCCTGGTACTC
	TCAGTTGGTGTGGGCATACT
Rank	CGACTGGTTCACTGCTCCTA
	GTGAAGTCACAGCCCTCAGA
Rankl	CGCAGATGGATCCTAACAGA
	CTGCAAATCTGCGTTTTCAT
Opg	CTGGTCATCAGCTCCTGTGT
	TGTCTTCCTCCTCACTGTGC
Ctsk	CAGCAGAGGTGTGTACTATG
	GCGTTGTTCTTATTCCGAGC
