# Supplementary material for: Comprehensive analysis of the skeletal phenotype in Chst14−/− mice: implications for dermatan sulfate in bone structure and strength
Source: Glycobiology. 2026 May 15;36(7):cwag037. doi: 10.1093/glycob/cwag037 (PMC13196589; doi:10.1093/glycob/cwag037)
Supplement: Supplementary_matrials_cwag037 [file supplementary_matrials_cwag037.zip › Supplementary_Table_S2_(Glyco_Revise).pdf]

Table S2. Tukey's multiple comparisons test (Figure 2A)

Gene expression (*Chst14*)

Comparison	Predicted (LS) mean diff.	95.00% CI of diff.	Adjusted P Value
12w:+/+ vs. 12w:-/-	0.9886	0.3425 to 1.635	0.0024
12w:+/+ vs. 52w:+/+	-0.4915	-1.108 to 0.1244	0.1437
12w:+/+ vs. 52w:-/-	0.9934	0.2390 to 1.748	0.0082
12w:-/- vs. 52w:+/+	1.48	0.8341 to 2.126	<0.0001
12w:-/- vs. 52w:-/-	0.004818	-0.7743 to 0.7840	>0.9999
52w:+/+ vs. 52w:-/-	1.485	0.7305 to 2.239	0.0002

Gene expression (*Chst11*)

Comparison	Predicted (LS) mean diff.	95.00% CI of diff.	Adjusted P Value
12w:+/+ vs. 12w:-/-	-0.0801	-0.4135 to 0.2533	0.9051
12w:+/+ vs. 52w:+/+	-0.08948	-0.4229 to 0.2440	0.8736
12w:+/+ vs. 52w:-/-	-0.006161	-0.3559 to 0.3436	>0.9999
12w:-/- vs. 52w:+/+	0.009375	-0.3241 to 0.3428	0.9998
12w:-/- vs. 52w:-/-	0.07394	-0.2758 to 0.4237	0.9325
52w:+/+ vs. 52w:-/-	0.08332	-0.2664 to 0.4330	0.9071

Gene expression (*Chst12*)

Comparison	Predicted (LS) mean diff.	95.00% CI of diff.	Adjusted P Value
12w:+/+ vs. 12w:-/-	-0.1156	-0.4468 to 0.2155	0.7614
12w:+/+ vs. 52w:+/+	-0.1318	-0.4630 to 0.1993	0.6823
12w:+/+ vs. 52w:-/-	0.008113	-0.3392 to 0.3554	0.9999
12w:-/- vs. 52w:+/+	0.01622	-0.3149 to 0.3474	0.999
12w:-/- vs. 52w:-/-	0.1237	-0.2236 to 0.4710	0.7503
52w:+/+ vs. 52w:-/-	0.14	-0.2074 to 0.4873	0.6743
