# Supplementary material for: Comprehensive analysis of the skeletal phenotype in Chst14−/− mice: implications for dermatan sulfate in bone structure and strength
Source: Glycobiology. 2026 May 15;36(7):cwag037. doi: 10.1093/glycob/cwag037 (PMC13196589; doi:10.1093/glycob/cwag037)
Supplement: Supplementary_matrials_cwag037 [file supplementary_matrials_cwag037.zip › Supplementary Table S3 (Glyco Revise).pdf]

Table S3. Tukey's multiple comparisons test (Figure 5A)

Maximum load (N)

Comparison	Predicted (LS) mean diff.	95.00% CI of diff.	Adjusted P Value
12w:+/+ vs. 12w:-/-	1.379	-1.236 to 3.994	0.4794
12w:+/+ vs. 52w:+/+	-7.059	-9.976 to -4.142	<0.0001
12w:+/+ vs. 52w:-/-	-2.363	-5.280 to 0.5543	0.1426
12w:-/- vs. 52w:+/+	8.437	5.271 to 11.60	<0.0001
12w:-/- vs. 52w:-/-	-3.741	-6.908 to -0.5746	0.0164
52w:+/+ vs. 52w:-/-	4.696	1.275 to 8.117	0.0047

Breaking displacement (mm)

Comparison	Predicted (LS) mean diff.	95.00% CI of diff.	Adjusted P Value
12w:+/+ vs. 12w:-/-	0.006234	-0.1751 to 0.1876	0.9997
12w:+/+ vs. 52w:+/+	0.1911	-0.01125 to 0.3934	0.069
12w:+/+ vs. 52w:-/-	0.2431	0.04075 to 0.4454	0.0144
12w:-/- vs. 52w:+/+	-0.1849	-0.4045 to 0.03480	0.1211
12w:-/- vs. 52w:-/-	0.2369	0.01720 to 0.4565	0.0312
52w:+/+ vs. 52w:-/-	0.052	-0.1853 to 0.2893	0.9296

Stiffness (N/mm)

Comparison	Predicted (LS) mean diff.	95.00% CI of diff.	Adjusted P Value
12w:+/+ vs. 12w:-/-	21.95	6.605 to 37.29	0.0032
12w:+/+ vs. 52w:+/+	-35.89	-53.01 to -18.78	<0.0001
12w:+/+ vs. 52w:-/-	-28.95	-46.07 to -11.83	0.0005
12w:-/- vs. 52w:+/+	57.84	39.26 to 76.43	<0.0001
12w:-/- vs. 52w:-/-	-50.9	-69.48 to -32.31	<0.0001
52w:+/+ vs. 52w:-/-	6.946	-13.13 to 27.02	0.7759

Breaking energy (N.mm)

Comparison	Predicted (LS) mean diff.	95.00% CI of diff.	Adjusted P Value
12w:+/+ vs. 12w:-/-	0.463	-1.642 to 2.568	0.929
12w:+/+ vs. 52w:+/+	2.505	0.1559 to 4.853	0.0336
12w:+/+ vs. 52w:-/-	3.683	1.334 to 6.031	0.0012
12w:-/- vs. 52w:+/+	-2.042	-4.591 to 0.5081	0.1495
12w:-/- vs. 52w:-/-	3.22	0.6699 to 5.769	0.0097
52w:+/+ vs. 52w:-/-	1.178	-1.576 to 3.932	0.6449
