# Supplementary material for: Comprehensive analysis of the skeletal phenotype in Chst14−/− mice: implications for dermatan sulfate in bone structure and strength
Source: Glycobiology. 2026 May 15;36(7):cwag037. doi: 10.1093/glycob/cwag037 (PMC13196589; doi:10.1093/glycob/cwag037)
Supplement: Supplementary_matrials_cwag037 [file supplementary_matrials_cwag037.zip › Supplementary Table S4 (Glyco Revise).pdf]

Table S4. Tukey's multiple comparisons test (Figure 6A)

Serum Ca (mg/dL)

Comparison	Predicted (LS) mean diff.	95.00% CI of diff.	Adjusted P Value
12w:+/+ vs. 12w:-/-	-0.1	-0.5828 to 0.3828	0.937
12w:+/+ vs. 52w:+/+	-0.15	-0.6328 to 0.3328	0.8203
12w:+/+ vs. 52w:-/-	-0.03333	-0.5161 to 0.4494	0.9974
12w:-/- vs. 52w:+/+	0.05	-0.4328 to 0.5328	0.9912
12w:-/- vs. 52w:-/-	0.06667	-0.4161 to 0.5494	0.9798
52w:+/+ vs. 52w:-/-	0.1167	-0.3661 to 0.5994	0.9048

Serum IP (mg/dL)

Comparison	Predicted (LS) mean diff.	95.00% CI of diff.	Adjusted P Value
12w:+/+ vs. 12w:-/-	0.8333	-1.735 to 3.401	0.8007
12w:+/+ vs. 52w:+/+	1.283	-1.285 to 3.851	0.5146
12w:+/+ vs. 52w:-/-	1.1	-1.468 to 3.668	0.6346
12w:-/- vs. 52w:+/+	-0.45	-3.018 to 2.118	0.9603
12w:-/- vs. 52w:-/-	0.2667	-2.301 to 2.835	0.9912
52w:+/+ vs. 52w:-/-	-0.1833	-2.751 to 2.385	0.9971

Serum estrasiol (pg/mL)

Comparison	Predicted (LS) mean diff.	95.00% CI of diff.	Adjusted P Value
12w:+/+ vs. 12w:-/-	9.493	-9.259 to 28.25	0.5038
12w:+/+ vs. 52w:+/+	6.282	-12.47 to 25.03	0.7852
12w:+/+ vs. 52w:-/-	9.61	-9.142 to 28.36	0.4936
12w:-/- vs. 52w:+/+	3.212	-15.54 to 21.96	0.9628
12w:-/- vs. 52w:-/-	0.1167	-18.64 to 18.87	>0.9999
52w:+/+ vs. 52w:-/-	3.328	-15.42 to 22.08	0.9589
