# Supplementary material for: Comprehensive analysis of the skeletal phenotype in Chst14−/− mice: implications for dermatan sulfate in bone structure and strength
Source: Glycobiology. 2026 May 15;36(7):cwag037. doi: 10.1093/glycob/cwag037 (PMC13196589; doi:10.1093/glycob/cwag037)
Supplement: Supplementary_matrials_cwag037 [file supplementary_matrials_cwag037.zip › Supplementary Table S5 (Glyco Revise).pdf]

Table S5. Tukey's multiple comparisons test (Figure 6B)

Bone Ca (mg/g)

Comparison	Predicted (LS) mean diff.	95.00% CI of diff.	Adjusted P Value
12w:+/+ vs. 12w:-/-	2	-13.10 to 17.10	0.9727
12w:+/+ vs. 52w:+/+	-10	-25.10 to 5.096	0.2252
12w:+/+ vs. 52w:-/-	-16	-31.10 to -0.9040	0.0382
12w:-/- vs. 52w:+/+	12	-3.096 to 27.10	0.1261
12w:-/- vs. 52w:-/-	-18	-33.10 to -2.904	0.0213
52w:+/+ vs. 52w:-/-	-6	-21.10 to 9.096	0.6029

Bone P (mg/g)

Comparison	Predicted (LS) mean diff.	95.00% CI of diff.	Adjusted P Value
12w:+/+ vs. 12w:-/-	1	-4.748 to 6.748	0.942
12w:+/+ vs. 52w:+/+	-1.667	-7.415 to 4.082	0.7911
12w:+/+ vs. 52w:-/-	-5	-10.75 to 0.7484	0.0901
12w:-/- vs. 52w:+/+	2.667	-3.082 to 8.415	0.4875
12w:-/- vs. 52w:-/-	-6	-11.75 to -0.2516	0.0411
52w:+/+ vs. 52w:-/-	-3.333	-9.082 to 2.415	0.3166
