# Supplementary material for: Comprehensive analysis of the skeletal phenotype in Chst14−/− mice: implications for dermatan sulfate in bone structure and strength
Source: Glycobiology. 2026 May 15;36(7):cwag037. doi: 10.1093/glycob/cwag037 (PMC13196589; doi:10.1093/glycob/cwag037)
Supplement: Supplementary_matrials_cwag037 [file supplementary_matrials_cwag037.zip › Supplementary Table S6 (Glyco Revise).pdf]

Table S6. Tukey's multiple comparisons test (Figure 6E)

Gene expression (*Col1a1*)

Comparison	Predicted (LS) mean diff.	95.00% CI of diff.	Adjusted P Value
12w:+/+ vs. 12w:-/-	-0.2024	-0.8641 to 0.4594	0.8251
12w:+/+ vs. 52w:+/+	0.6329	-0.02885 to 1.295	0.0639
12w:+/+ vs. 52w:-/-	0.641	-0.05301 to 1.335	0.0765
12w:-/- vs. 52w:+/+	-0.8353	-1.497 to -0.1735	0.0105
12w:-/- vs. 52w:-/-	0.8434	0.1494 to 1.537	0.0141
52w:+/+ vs. 52w:-/-	0.008142	-0.6859 to 0.7022	>0.9999

Gene expression (*Dcn*)

Comparison	Predicted (LS) mean diff.	95.00% CI of diff.	Adjusted P Value
12w:+/+ vs. 12w:-/-	-0.07068	-0.7097 to 0.5683	0.9892
12w:+/+ vs. 52w:+/+	0.4505	-0.1884 to 1.090	0.2291
12w:+/+ vs. 52w:-/-	0.5764	-0.09378 to 1.247	0.1073
12w:-/- vs. 52w:+/+	-0.5212	-1.160 to 0.1178	0.1348
12w:-/- vs. 52w:-/-	0.6471	-0.02310 to 1.317	0.0607
52w:+/+ vs. 52w:-/-	0.1258	-0.5443 to 0.7960	0.9512

Gene expression (*Bgn*)

Comparison	Predicted (LS) mean diff.	95.00% CI of diff.	Adjusted P Value
12w:+/+ vs. 12w:-/-	-0.2429	-0.9130 to 0.4272	0.7404
12w:+/+ vs. 52w:+/+	0.252	-0.4181 to 0.9221	0.7186
12w:+/+ vs. 52w:-/-	0.2428	-0.4600 to 0.9455	0.7671
12w:-/- vs. 52w:+/+	-0.4949	-1.165 to 0.1752	0.1964
12w:-/- vs. 52w:-/-	0.4857	-0.2171 to 1.188	0.2441
52w:+/+ vs. 52w:-/-	-0.009244	-0.7120 to 0.6936	>0.9999
