# Supplementary material for: Comprehensive analysis of the skeletal phenotype in Chst14−/− mice: implications for dermatan sulfate in bone structure and strength
Source: Glycobiology. 2026 May 15;36(7):cwag037. doi: 10.1093/glycob/cwag037 (PMC13196589; doi:10.1093/glycob/cwag037)
Supplement: Supplementary_matrials_cwag037 [file supplementary_matrials_cwag037.zip › Supplementary Table S7 (Glyco Revise).pdf]

Table S7. Tukey's multiple comparisons test (Figure 7A)

Gene expression (*Bglap*)

Comparison	Predicted (LS) mean diff.	95.00% CI of diff.	Adjusted P Value
12w:+/+ vs. 12w:-/-	-0.272	-0.9840 to 0.4400	0.7089
12w:+/+ vs. 52w:+/+	0.5783	-0.1337 to 1.290	0.1372
12w:+/+ vs. 52w:-/-	0.6412	-0.1055 to 1.388	0.1081
12w:-/- vs. 52w:+/+	-0.8503	-1.562 to -0.1383	0.016
12w:-/- vs. 52w:-/-	0.9132	0.1665 to 1.660	0.0134
52w:+/+ vs. 52w:-/-	0.06298	-0.6837 to 0.8097	0.9951

Gene expression (*Acp5*)

Comparison	Predicted (LS) mean diff.	95.00% CI of diff.	Adjusted P Value
12w:+/+ vs. 12w:-/-	-0.2002	-1.008 to 0.6080	0.8971
12w:+/+ vs. 52w:+/+	-0.5825	-1.391 to 0.2257	0.2133
12w:+/+ vs. 52w:-/-	-1.364	-2.211 to -0.5161	0.0012
12w:-/- vs. 52w:+/+	0.3823	-0.4259 to 1.190	0.5561
12w:-/- vs. 52w:-/-	-1.164	-2.011 to -0.3159	0.0053
52w:+/+ vs. 52w:-/-	-0.7813	-1.629 to 0.06637	0.0773
