# Supplementary material for: Comprehensive analysis of the skeletal phenotype in Chst14−/− mice: implications for dermatan sulfate in bone structure and strength
Source: Glycobiology. 2026 May 15;36(7):cwag037. doi: 10.1093/glycob/cwag037 (PMC13196589; doi:10.1093/glycob/cwag037)
Supplement: Supplementary_matrials_cwag037 [file supplementary_matrials_cwag037.zip › Supplementary Table S8 (Glyco Revise).pdf]

Table S8. Tukey's multiple comparisons test (Figure 7B)

Gene expression (*Rank*)

Comparison	Predicted (LS) mean diff.	95.00% CI of diff.	Adjusted P Value
12w:+/+ vs. 12w:-/-	-0.2623	-1.520 to 0.9956	0.935
12w:+/+ vs. 52w:+/+	-0.1128	-1.371 to 1.145	0.9942
12w:+/+ vs. 52w:-/-	-1.728	-3.047 to -0.4087	0.0079
12w:-/- vs. 52w:+/+	-0.1495	-1.407 to 1.108	0.9867
12w:-/- vs. 52w:-/-	-1.466	-2.785 to -0.1463	0.0263
52w:+/+ vs. 52w:-/-	-1.615	-2.935 to -0.2958	0.0133

Gene expression (*Rankl*)

Comparison	Predicted (LS) mean diff.	95.00% CI of diff.	Adjusted P Value
12w:+/+ vs. 12w:-/-	-0.548	-1.693 to 0.5964	0.5462
12w:+/+ vs. 52w:+/+	-0.08433	-1.229 to 1.060	0.9967
12w:+/+ vs. 52w:-/-	-0.7991	-1.999 to 0.4012	0.2726
12w:-/- vs. 52w:+/+	-0.4637	-1.608 to 0.6808	0.6706
12w:-/- vs. 52w:-/-	-0.2511	-1.451 to 0.9492	0.9344
52w:+/+ vs. 52w:-/-	-0.7148	-1.915 to 0.4855	0.3636

Gene expression (*Opg*)

Comparison	Predicted (LS) mean diff.	95.00% CI of diff.	Adjusted P Value
12w:+/+ vs. 12w:-/-	-0.1232	-1.071 to 0.8242	0.9828
12w:+/+ vs. 52w:+/+	-0.3259	-1.273 to 0.6215	0.7693
12w:+/+ vs. 52w:-/-	-0.7339	-1.728 to 0.2598	0.1964
12w:-/- vs. 52w:+/+	0.2027	-0.7447 to 1.150	0.9303
12w:-/- vs. 52w:-/-	-0.6107	-1.604 to 0.3830	0.3373
52w:+/+ vs. 52w:-/-	-0.408	-1.402 to 0.5857	0.6615
