# Supplementary material for: Comprehensive analysis of the skeletal phenotype in Chst14−/− mice: implications for dermatan sulfate in bone structure and strength
Source: Glycobiology. 2026 May 15;36(7):cwag037. doi: 10.1093/glycob/cwag037 (PMC13196589; doi:10.1093/glycob/cwag037)
Supplement: Supplementary_matrials_cwag037 [file supplementary_matrials_cwag037.zip › Supplementary Table S9 (Glyco Revise).pdf]

Table S9. Tukey's multiple comparisons test (Figure 7C and D)

Gene expression (*Ctsk*)

Comparison	Predicted (LS) mean diff.	95.00% CI of diff.	Adjusted P Value
12w:+/+ vs. 12w:-/-	-0.07299	-0.8679 to 0.7220	0.9938
12w:+/+ vs. 52w:+/+	-0.06821	-0.8632 to 0.7267	0.9949
12w:+/+ vs. 52w:-/-	-0.6344	-1.468 to 0.1994	0.1766
12w:-/- vs. 52w:+/+	-0.004777	-0.7997 to 0.7902	>0.9999
12w:-/- vs. 52w:-/-	-0.5614	-1.395 to 0.2723	0.2638
52w:+/+ vs. 52w:-/-	-0.5662	-1.400 to 0.2676	0.2573

TRACP-5b (U/L)

Comparison	Predicted (LS) mean diff.	95.00% CI of diff.	Adjusted P Value
12w:+/+ vs. 12w:-/-	0.9	-0.9545 to 2.754	0.5385
12w:+/+ vs. 52w:+/+	0.8333	-1.021 to 2.688	0.599
12w:+/+ vs. 52w:-/-	1.3	-0.5545 to 3.154	0.2352
12w:-/- vs. 52w:+/+	-0.06667	-1.921 to 1.788	0.9996
12w:-/- vs. 52w:-/-	0.4	-1.454 to 2.254	0.9297
52w:+/+ vs. 52w:-/-	0.4667	-1.388 to 2.321	0.8942
