# Supplementary material for: Comprehensive analysis of the skeletal phenotype in Chst14−/− mice: implications for dermatan sulfate in bone structure and strength
Source: Glycobiology. 2026 May 15;36(7):cwag037. doi: 10.1093/glycob/cwag037 (PMC13196589; doi:10.1093/glycob/cwag037)
Supplement: Supplementary_matrials_cwag037 [file supplementary_matrials_cwag037.zip › Supplementary Table S10 (Glyco Revise).pdf]

Table S10. Tukey's multiple comparisons test (Figure 8B-D)

B) BV/TV(%)

Comparison	Predicted (LS) mean diff.	95.00% CI of diff.	Adjusted P Value
12w:+/+ vs. 12w:-/-	3.979	0.3575 to 7.600	0.0321
12w:+/+ vs. 52w:+/+	6.608	2.986 to 10.23	0.0017
12w:+/+ vs. 52w:-/-	6.572	2.951 to 10.19	0.0018
12w:-/- vs. 52w:+/+	2.629	-0.9922 to 6.250	0.1712
12w:-/- vs. 52w:-/-	2.593	-1.028 to 6.215	0.1787
52w:+/+ vs. 52w:-/-	-0.03567	-3.657 to 3.586	>0.9999

C) Tb.N (N/mm)

Comparison	Predicted (LS) mean diff.	95.00% CI of diff.	Adjusted P Value
12w:+/+ vs. 12w:-/-	1.133	0.04527 to 2.221	0.0415
12w:+/+ vs. 52w:+/+	1.92	0.8323 to 3.008	0.0021
12w:+/+ vs. 52w:-/-	1.94	0.8523 to 3.028	0.002
12w:-/- vs. 52w:+/+	0.787	-0.3011 to 1.875	0.1732
12w:-/- vs. 52w:-/-	0.807	-0.2811 to 1.895	0.1598
52w:+/+ vs. 52w:-/-	0.02	-1.068 to 1.108	>0.9999

D) Tb/Th (µm)

Comparison	Predicted (LS) mean diff.	95.00% CI of diff.	Adjusted P Value
12w:+/+ vs. 12w:-/-	2.041	-15.40 to 19.48	0.9808
12w:+/+ vs. 52w:+/+	2.325	-15.11 to 19.76	0.9722
12w:+/+ vs. 52w:-/-	1.774	-15.66 to 19.21	0.9872
12w:-/- vs. 52w:+/+	0.2837	-17.15 to 17.72	>0.9999
12w:-/- vs. 52w:-/-	-0.267	-17.70 to 17.17	>0.9999
52w:+/+ vs. 52w:-/-	-0.5507	-17.99 to 16.89	0.9996
