# Supplementary material for: Comprehensive analysis of the skeletal phenotype in Chst14−/− mice: implications for dermatan sulfate in bone structure and strength
Source: Glycobiology. 2026 May 15;36(7):cwag037. doi: 10.1093/glycob/cwag037 (PMC13196589; doi:10.1093/glycob/cwag037)
Supplement: Supplementary_matrials_cwag037 [file supplementary_matrials_cwag037.zip › Supplementary Table S11 (Glyco Revise).pdf]

Table S11. Tukey's multiple comparisons test (Figure S2A)

N.Oc/BS (N/mm)

Comparison	Predicted (LS) mean diff.	95.00% CI of diff.	Adjusted P Value
12w:+/+ vs. 12w:-/-	-0.2667	-1.749 to 1.215	0.9365
12w:+/+ vs. 52w:+/+	-0.3617	-1.844 to 1.120	0.8608
12w:+/+ vs. 52w:-/-	0.3227	-1.159 to 1.805	0.8954
12w:-/- vs. 52w:+/+	-0.095	-1.577 to 1.387	0.9967
12w:-/- vs. 52w:-/-	0.5893	-0.8927 to 2.071	0.6025
52w:+/+ vs. 52w:-/-	0.6843	-0.7977 to 2.166	0.4911

Oc.S/BS (%)

Comparison	Predicted (LS) mean diff.	95.00% CI of diff.	Adjusted P Value
12w:+/+ vs. 12w:-/-	-1.332	-5.110 to 2.446	0.6834
12w:+/+ vs. 52w:+/+	-0.8907	-4.669 to 2.888	0.8722
12w:+/+ vs. 52w:-/-	0.9307	-2.848 to 4.709	0.8576
12w:-/- vs. 52w:+/+	0.4413	-3.337 to 4.220	0.9809
12w:-/- vs. 52w:-/-	2.263	-1.516 to 6.041	0.2934
52w:+/+ vs. 52w:-/-	1.821	-1.957 to 5.600	0.4578
